# Rational Design and Synthesis of 3-Morpholine Linked Aromatic-Imino-1*H*-Indoles as Novel Kv1.5 Channel Inhibitors Sharing Vasodilation Effects

**DOI:** 10.3389/fmolb.2021.805594

**Published:** 2022-01-24

**Authors:** Wei Qin, Yi-Heng Li, Jing Tong, Jie Wu, Dong Zhao, Hui-Jin Li, Lu Xing, Chun-Xia He, Xin Zhou, Peng-Quan Li, Ge Meng, Shao-Ping Wu, Hui-Ling Cao

**Affiliations:** ^1^ Xi’an Key Laboratory of Basic and Translation of Cardiovascular Metabolic Disease, Shaanxi Key Laboratory of Ischemic Cardiovascular Disease, Institute of Basic and Translational Medicine, Xi’an Medical University, Xi’an, China; ^2^ College of Life Sciences, Northwest University, Xi’an, China; ^3^ School of Pharmacy, Health Science Center, Xi’an Jiaotong University, Xi’an, China; ^4^ Engineering Center of Catalysis and Synthesis for Chiral Molecules, Shanghai Engineering Center of Industrial Asymmetric Catalysis for Chiral Drugs, Fudan University, Shanghai, China

**Keywords:** Kv1.5 inhibitor, drug design, anti-atrial fibrillation, anti-hypertension, lead compound

## Abstract

Atrial fibrillation (AF) is the most common clinical sustained arrhythmia; clinical therapeutic drugs have low atrial selectivity and might cause more severe ventricle arrhythmias while stopping AF. As an anti-AF drug target with high selectivity on the atrial muscle cells, the undetermined crystal structure of Kv1.5 potassium channel impeded further new drug development. Herein, with the simulated 3D structure of Kv1.5 as the drug target, a series of 3-morpholine linked aromatic amino substituted 1*H*-indoles as novel Kv1.5 channel inhibitors were designed and synthesized based on target–ligand interaction analysis. The synthesis route was practical, starting from commercially available material, and the chemical structures of target compounds were characterized. It was indicated that compounds **T16** and **T5** (100 μM) exhibited favorable inhibitory activity against the Kv1.5 channel with an inhibition rate of 70.8 and 57.5% using a patch clamp technique. All compounds did not exhibit off-target effects against other drug targets, which denoted some selectivity on the Kv1.5 channel. Interestingly, twelve compounds exhibited favorable vasodilation activity on pre-contracted arterial rings *in vitro* using KCl or phenylephrine (PE) by a Myograph. The vasodilation rates of compounds **T16** and **T4** (100 μM) even reached over 90%, which would provide potential lead compounds for both anti-AF and anti-hypertension new drug development.

## Highlights

1. Sixteen 3-morpholine linked aromatic amino substituted 1*H*-indoles as novel Kv1.5 channel inhibitors were designed and synthesized targeting the simulated 3D structure of Kv1.5.
2. Compounds **T16** and **T5** (100 μM) exhibited favorable inhibitory activity against the Kv1.5 channel with an inhibition rate of 70.8 and 57.5% using a patch clamp technique. The vasodilation rates of compounds **T16** and **T4** (100 μM) reached over 90% by a Myograph.
3. The novel Kv1.5 inhibitors would provide potential lead compounds for both anti-atrial fibrillation and anti-hypertension new drug development.

## 1 Introduction

As the most common clinical sustained arrhythmia, atrial fibrillation (AF) presents with a wide spectrum of symptoms and severity (Lau et al., 2019). There are now many therapeutic drugs for clinically treating AF, such as Dofetilide, Aminodarone, Sotalol, Propafenone, flecainide, etc. These drugs have low atrial selectivity while stopping AF, which could often cause severe adverse effects such as ventricle arrhythmias (e.g., life-threatening TdP) (Ang et al., 2020). Therefore, an ideal drug to treat AF should have high selectivity on the atrial muscle cells, which could terminate or postpone the occurrence of AF without affecting or prolonging the Q-T interval or the negative inotropic action to ensure the highly effective safety (Guo et al., 2016; Zimetbaum, 2017).

The cardiac I_Kur_ (Kv1.5) potassium channel is a kind of voltage-gated ion-channel which has been confirmed to be expressed only in the human atrium (Wettwer and Terlau, 2014; Du et al., 2021). Therefore, Kv1.5 is currently considered as the ideal target for designing the highly atrium-selective drugs to treat AF (Guo et al., 2012; Zhao et al., 2019). Besides, the clinical and animal research data showed that the Kv1.5 channel is the base for the atrium-reconstructing electric rhythm. The inhibitor of the Kv1.5 channel might selectively prolong the effective refractory period (ERP) of the atrium to terminate AF (Tamargo et al., 2009).

**FIGURE 1:**
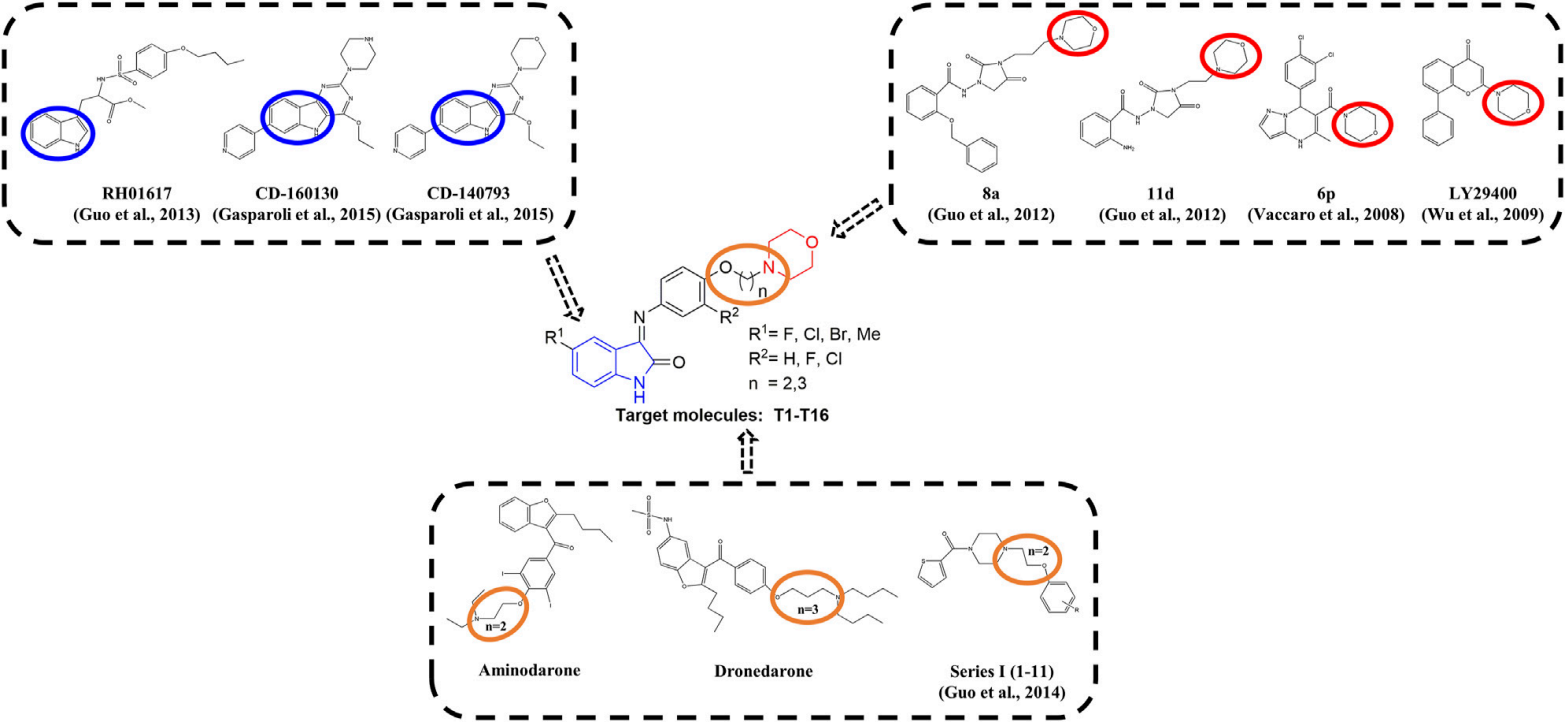
Design strategy of novel Kv1.5 inhibitors based on morpholine, 1*H*-indole, and the active linker (-N(CH_2_)_n_O-).

Multiple studies indicated that some compounds with the skeleton of morpholine exhibited inhibitive effects against Kv1.5 (Vaccaro et al., 2008; Guo et al., 2012), such as the compound LY294002 (Wu et al., 2009; Hong et al., 2013) (Figure 1). Some of the chemical structures with 1*H*-indole (Guo et al., 2013) or pyrimidine fused 1*H*-indole skeletons (Gasparoli et al., 2015) also showed inhibitive activity on the Kv1.5 channel (Figure 1). Sharing the common structure features, the two compounds (CD-160130 and CD-140793) contained both morpholine and 1*H*-indole functional groups, which were connected by several chemical bonds within a pyrimidine ring (Figure 1). The distance between morpholine and 1*H*-indole in the chemical structures are in a certain scope. To be specific, the distance between N on morpholine and 3-C on indole in these compounds are from 2–3 atoms to 3–4 bond length (Figure 1). The clinical drugs including Aminodarone and Dronedarone are used as the anti-arrhythmic drugs (Figure 1). Aminodarone might lengthen the monophasic action potential duration to prolong the ERP (Mujović et al., 2020). Dronedarone is a kind of multichannel-blocker with the electronic physiological and blood dynamic features similar to Aminodarone. Dronedarone could be used to prevent AF and ventricular tachycadia and restore the sinus rhythm (Kaess and Ehrlich, 2016). The common chemical structure feature of the two drugs is the linker between nitrogen and oxygen, that is, -N(CH_2_)_n_O- (*n* = 2–3). The chain-like linker is exactly alike to the structure feature of other kinds of Kv1.5 inhibitors (Guo et al., 2014) (Figure 1).

As an anti-AF drug target with high selectivity on the atrial muscle cells, the undetermined crystal structure of the Kv1.5 channel impeded further new drug development. Herein, the simulated 3D structure of the Kv1.5 channel as the drug target was built based on the crystal structure of its homolog Kv1.2 channel and its amino acid sequence. Based on target–ligand interaction and structure–activity analysis of traditional inhibitors as well as its simulated 3D structure of target Kv1.5, a series of compounds (T1–T16), containing both morpholine and 1*H*-indole linked with a chain-like structure (-N(CH_2_)_n_O-) in one molecule, were designed and synthesized to enhance therapeutic effects of the antiarrhythmic drugs (Figure 1).

## 2 Materials and Methods

### 2.1 Chemistry

#### 2.1.1 General Procedures to Prepare 4-Substituted-N-Hydroxyimino Acetyl Aniline (2a-2d) (Moreira et al., 2012)

Chloral hydrate (9.0 g, 55.0 mmol, 1.1 equiv) and water (240 ml) were added to a clean 500-ml flask under stirring, to which was added anhydrous sodium sulfate (65.0 g), substituted aniline (**1a-1d**, 50.0 mmol, 1.0 equiv), hydrochloride solution (4.3 ml HCl +30.0 ml water), and hydroxylamine hydrochloride solution (10.8 g + 50.0 ml water), under stirring sequentially. The reaction was heated gradually to 65°C for 2 h before heating was stopped. The hot mixture was filtered to give a clear solution, from which some pale yellow solids were precipitated while it was being cooled down to room temperature. The filtration under the reduced pressure could yield the desired product (**2a-2d**) (petroleum ether/ethyl acetate, 1:2, R_f_ = 0.2–0.4).

#### 2.1.2 General Procedures to Prepare 5-Substituted-isatin (3a-3d)

Concentrated sulfuric acid (24.0 ml) was added to a clean 150-ml three-neck flask. The reaction mixture was heated to 50°C, to which was added the intermediate **2a-2d** (30.0 mmol, 1.0 equiv) obtained from the above procedure. The color of the reaction mixture became dark to dark-black, while the amount of the material was added increasingly. The temperature of the reaction mixture was adjusted to 80°C for 20 min. The crashed ice (100 g) was added slowly to the reaction system to allow it to turn a red-brown color. The reaction mixture was kept at room temperature overnight before being filtrated to produce the filter cake, which was washed to pH = 7 with water to yield a solid. The solid was dissolved in an aqueous solution of NaOH (10.0%, 90.0 ml) before being filtrated. The pH of the filtration was adjusted to pH = 2 with concentrated hydrochloride until there were a large amount of brick-red solids precipitated from the solution. The solid was filtered to yield the red-brown solid as the desired products (**3a-3d**, petroleum ether/ethyl acetate, 1:2, R_f_ = 0.4–0.6).

#### 2.1.3 General Procedures for the Synthesis of Target Compounds T1-T16

##### 2.1.3.1 5-Fluoro-3-[(4-(2-Morpholine-4-yl-Ethoxyl)Phenyl)Imino]-1,3-Dihydro-1H-Indole-2-One(T1)

5-Fluoro-isatin (**3a**, 0.340 g, 2.00 mmol, 1.0 equiv) and 1-(2-(4-aminophenyloxyl)ethyl) morpholine (**4a**, 0.444 g, 2.00 mmol, 1.0 equiv) were added to a 50-ml flask, to which was added absolute ethanol (10.0 ml) and acetic acid (1–2 drops) at room temperature under stirring. The reaction mixture was heated to refluxing for 10 h, before being monitored by thin layer chromatography (TLC) until the reaction was completed before being cooled down to room temperature. A large amount of yellow solid precipitated, which was collected by filtering under reduced pressure to yield the yellow powder as the desired target compound (**T1**, 0.442 g, 60%). m. p. 155–157 °C (dichloromethane/methanol, 10:1, R_f_ = 0.4), ^1^H NMR (400 MHz, DMSO-*d*_6_) *δ*: ^1^H NMR (400 MHz, DMSO-*d*_6_) *δ*: 10.95 (d, *J* = 8.4 Hz, 1H), 7.25 (t, *J* = 8.3 Hz, 2H), 7.08 (d, *J* = 8.4 Hz, 1H), 7.00 (d, *J* = 8.4 Hz, 1H), 6.95–6.82 (m, 1H), 6.34 (d, *J* = 7.4 Hz, 1H), 4.18–4.10 (m, 2H), 3.59 (s, 4H), 2.72 (d, *J* = 5.0 Hz, 2H). HRMS (ESI): *m/z* calculated for C_20_H_20_FN_3_O_3_ 369.3895, Found 369.3895.

##### 2.1.3.2 5-Fluoro-3-[(3-Fluoro-4-(3-Morpholine-4-yl-Propoxyl)Phenyl)Imino]-1,3-Dihydro-1H-Indole-2-One(T2)

5-Fluoro-isatin (3a, 0.34 g, 2.0 mmol, 1.0 equiv) and 1-[2-(2-fluoro-4-amino-phenoxy) propyl] morpholine (**4b**, 0.508 g, 2.00 mmol, 1.0 equiv) were added to a 50-ml flask, to which was added absolute ethanol (10.0 ml) and acetic acid (1–2 drops) at room temperature under stirring. The reaction mixture was heated to refluxing for 13 h before being completed as shown by TLC analysis to ensure the reaction completion. The reaction mixture was cooled down to room temperature without any solid being precipitated. A certain amount of water was added into the reaction system to allow the large amount of red solid to be precipitated. The solids were collected *via* filtration under the reduced pressure and then dried under *vacuo* to yield the red solids as the desired target compound (**T2**, 0.150 g, 19%). m. p. 131–133°C (dichloromethane/methanol, 10:1, R_f_ = 0.5), ^1^H NMR (400 MHz, DMSO-*d*_6_) *δ*: 10.97 (d, *J* = 8.0 Hz, 1H), 7.28 (dd, *J* = 8.4, 7.5 Hz, 2H), 7.22–7.10 (m, 1H), 7.04 (dd, *J* = 7.0, 1.8 Hz, 1H), 6.99–6.89 (m, 1H), 6.85 (dd, *J* = 8.3, 6.2 Hz, 1H), 6.33–6.28 (m, 1H), 4.12 (dd, *J* = 5.4, 6.3 Hz, 2H), 3.58 (s, 4H), 2.48–2.36 (m, 6H), 1.92 (dd, *J* = 3.1, 6.6 Hz, 2H). HRMS (ESI): *m/z* calculated for C_21_H_21_F_2_N_3_O_3_ 401.4065, Found 401.4066.

##### 2.1.3.3 5-Fluoro-3-[(4-(3-Morpholine-4-yl-Propoxyphenyl)Imino]-1,3-Dihydro-1H-Indole-2-One(T3)

5-Fluoro-isatin (**3a**, 0.340 g, 2.00 mmol, 1.0 equiv) and 1-[2-(4-amino-phenyloxyl)propyl] morpholine (**4c**, 0.470 g, 2.00 mmol, 1.0 equiv) were added to a 50-ml flask, to which was added absolute ethanol (10.0 ml) and acetic acid (1–2 drops) under stirring at room temperature. The reaction mixture was heated to reflux for 10 h, which was monitored by TLC analysis to ensure the completed reaction. The reaction system was then allowed to be cooled down to room temperature without the precipitated solid. A small amount of water was then added to this system to allow a large amount of orange-yellow solid to be precipitated. The solid was obtained by filtration to give the orange-yellow powder as the desired target compound (**T3**, 0.389 g, 51%). m. p. 163–165°C (dichloromethane/methanol, 10:1, R_f_ = 0.4), ^1^H NMR (400 MHz, DMSO-*d*_6_) *δ*: 11.01 (s, 1H), 7.24 (dd, *J* = 8.6, 6.1, 2.7 Hz, 1H), 7.06 (d, *J* = 8.8 Hz, 2H), 7.00 (d, *J* = 8.8 Hz, 2H), 6.91 (dd, *J* = 8.2, 4.9 Hz, 1H), 6.34 (dd, *J* = 8.5, 2.3 Hz, 1H), 4.05 (dt, *J* = 2.1, 6.2 Hz, 2H), 3.59 (s, 4H), 2.48 (s, 1H), 2.47 (s, 1H), 2.42 (s, 4H), 1.91 (dd, *J* = 3.4, 6.6 Hz, 2H). HRMS (ESI): *m/z* calculated for C_21_H_22_FN_3_O_3_ 383.4161, Found 383.4162.

##### 2.1.3.4 3-[(3-Chloro-4-(3-Morpholine-4-ylPropoxy)Phenyl)Imino]-5-Fluoro-1,3-Dihydro-1H-Indole-2-One(T4)

5-Fluoro-isatin (**3a**, 0.340 g, 1.00 mmol, 1.0 equiv) and 1-[2-(2-chloro-4-aminophenoxyl) propyl] morpholine (**4d**, 0.540 g, 2.00 mmol, 2.0 equiv) were added to a 50-ml flask, to which was added absolute ethanol (10.0 ml) and acetic acid (1–2 drops) under stirring at room temperature. The reaction system was heated to reflux for 10 h under stirring until the TLC analysis showed the reaction completion. The reaction system was then cooled down to room temperature, until there was a large amount of dark-red solid being precipitated, which was filtrated before it was dried under *vacuo* to produce the dark red solid as the target compound (**T4**, 0.428 g, 51%). m. p. 127–128°C (dichloromethane/methanol, 10:1, R_f_ = 0.4), ^1^H NMR (400 MHz, DMSO-*d*_6_) *δ*: 10.96 (dd, *J* = 8.3, 9.9 Hz, 1H), 7.41–7.34 (m, 1H), 7.31–7.19 (m, 1H), 7.17–7.10 (m, 2H), 7.02 (dd, *J* = 1.7, 8.8, 6.5 Hz, 1H), 6.95–6.83 (m, 1H), 6.32 (dd, *J* = 5.2, 8.5, 2.6 Hz, 1H), 4.15 (dd, *J* = 5.4, 5.2 Hz, 1H), 4.13–4.01 (m, 1H), 3.60 (s, 4H), 2.53 (dd, *J* = 3.1, 4.5 Hz, 3H), 2.44 (s, 3H), 1.94 (dd, *J* = 3.9, 7.1 Hz, 2H). HRMS (ESI): *m/z* calculated for C_21_H_21_ClFN_3_O_3_ 417.8611, Found 417.8610.

##### 2.1.3.5 5-Chloro-3-[(4-(2-Morpholine-4-yl-Ethoxyl)Phenyl)Imino]-1,3-Dihydro-2H-Indole-2-One(T5)

5-Chloro-isatin (**3b**, 0.360 g, 1.00 mmol, 1.0 equiv) and 1-[2-(4-aminophenoxyl)ethyl]morpholine (**4a**, 0.444 g, 2.00 mmol, 2.0 equiv) were added to a 50-ml flask, to which was added absolute ethanol (10.0 ml) and acetic acid (1–2 drops) at room temperature under stirring. The reaction mixture was heated to reflux for 10 h until the TLC analysis showed the reaction completion. The reaction system was then allowed to be cooled down to room temperature, when there was a large amount of orange-red solids being precipitated. The solid was obtained *via* filtration under the reduced pressure. The filter cake was dried under *vacuo* to provide the orange-red solid as the desired target compound (**T5**, 0.753 g, 98%). m. p. 150–152°C (dichloromethane/methanol, 10:1, R_f_ = 0.5) ^1^H NMR (400 MHz, DMSO-*d*_6_) *δ*: 11.07 (d, *J* = 8.9 Hz, 1H), 7.47–7.37 (m, 1H), 7.26 (d, *J* = 8.7 Hz, 1H), 7.09 (d, *J* = 8.7 Hz, 1H), 7.01 (d, *J* = 8.7 Hz, 1H), 6.90 (dd, *J* = 8.2, 8.3 Hz, 2H), 6.58 (d, *J* = 1.4 Hz, 1H), 4.19–4.08 (m, 2H), 3.59 (d, *J* = 4.1 Hz, 4H), 2.72 (t, *J* = 5.5 Hz, 2H). ^13^C NMR (400 MHz, DMSO-*d*_6_) HRMS (ESI): *m/z* calculated for C_20_H_20_ClN_3_O_3_ 385.8441, Found 385.8442.

##### 2.1.3.6 5-Chloro-3-[(3-Fluoro-4-(3-Morpholine-4-yl-Propyloxy)Phenyl)Imino]-1,3-Dihydro-1H-Indole-2-One(T6)

5-Chloro-isatin (**3b**, 0.360 g, 1.00 mmol, 1.0 equiv) and 1-[2-(2-fluoro-4-aminophenoxyl)propyl] morpholine (**4b**, 0.508 g, 2.00 mmol, 2.0 equiv) were added to a 50-ml flask, to which were added absolute ethanol (10.0 ml) and acetic acid (1–2 drops) at room temperature under stirring. The reaction mixture was then heated to reflux for 11 h until the TLC analysis showed the reaction completion. The reaction system was then allowed to cool down to room temperature until there was a large amount of dark red solid being precipitated. The solid was obtained *via* filtration under the reduced pressure. The filter cake was dried under *vacuo* to yield the red solid as the desired target compound (**T6**, 0.357 g, 64%). m. p. 148–149°C (dichloromethane/methanol, 10:1, R_f_ = 0.4), ^1^H NMR (400 MHz, DMSO-*d*_6_) *δ*: 11.09 (d, *J* = 8.4 Hz, 1H), 7.46 (dd, *J* = 8.8, 8.4, 8 Hz, 1H), 7.31 (t, *J* = 8.9 Hz, 1H), 7.24–7.11 (m, 1H), 7.05 (dd, *J* = 8.0, 2.4 Hz, 1H), 6.94 (d, *J* = 8.4 Hz, 1H), 6.52 (d, *J* = 2.1 Hz, 1H), 4.13 (dt, *J* = 8.0, 6.3 Hz, 2H), 3.58 (t, *J* = 4.5 Hz, 4H), 2.44 (dd, *J* = 7.0, 6.1 Hz, 2H), 2.38 (s, 4H), 1.90 (dd, *J* = 3.0, 6.5 Hz, 2H). HRMS (ESI): *m/z* calculated for C_21_H_21_ClFN_3_O_3_ 417.8611, Found 417.8612.

##### 2.1.3.7 5-Chloro-3-[(4-(3-Morpholine-4-yl-PropyloxyPhenyl)Imino]-1,3-Dihydro-1*H*-Indole-2-One(T7)

5-Chloro-isatin (**3b**, 0.360 g, 1.00 mmol, 1.0 equiv),1-[2-(4-aminophenoxyl)propyl]morpholine (**4c**, 0.470 g, 2.00 mmol, 2.0 equiv) were added to a 50-ml flask, to which was added absolute ethanol (10.0 ml) and acetic acid (1–2 drops) at room temperature under stirring. The reaction mixture was then heated to reflux for 11 h until the TLC analysis showed the reaction completion. The reaction system was then allowed to cool down to room temperature until there was a large amount of orange-red solid being precipitated. The solid was obtained *via* filtration under the reduced pressure. The filter cake was dried under *vacuo* to yield the orange-red solid as the desired target compound (**T7**, 0.560 g, 70%). m. p. 171–173°C (dichloromethane/methanol, 10:1, R_f_ = 0.4), ^1^H NMR (400 MHz, DMSO-*d*_6_) *δ*: 11.13 (s, 1H), 7.43 (dd, *J* = 8.7, 8.4 Hz, 1H), 7.26 (d, *J* = 8.8 Hz, 1H), 7.07 (d, *J* = 8.9 Hz, 2H), 7.01 (d, *J* = 8.8 Hz, 2H), 6.93 (d, *J* = 8.5 Hz, 1H), 6.90–6.85 (m, 1H), 6.59 (d, *J* = 2.1 Hz, 1H), 4.11–4.00 (m, 2H), 3.58 (t, *J* = 4.5 Hz, 4H), 2.51 (d, *J* = 1.6 Hz, 1H), 2.43 (dd, *J* = 3.4, 6.3 Hz, 2H), 2.38 (s, 4H), 1.97–1.83 (m, 2H). HRMS (ESI): *m/z* calculated for C_21_H_22_ClN_3_O_3_ 399.8707, Found 399.8709.

##### 2.1.3.8 3-[(3-Chloro-4-(3-morpholine-4-yl-propyloxy)phenyl)imino]-5-chloro-1,3-dihydro-1*H*-indole-2-one(T8)

5-Chloro-isatin (**3b**, 0.360 g, 1.00 mmol, 1.0 equiv) and 1-[2-(2-chloro-4- aminophenoxy)propyl] morpholine (**4d**, 0.540 g, 2.00 mmol, 2.0 equiv) were added to a 50-ml flask, to which was added absolute ethanol (10.0 ml) and acetic acid (1–2 drops) at room temperature under stirring. The reaction mixture was heated to reflux for 11 h until the TLC analysis showed that the reaction was completed. The reaction system was then allowed to cool down to room temperature until there was a large amount of dark red solid being precipitated. The solids were obtained *via* filtration under the reduced pressure. The filter cake was dried under *vacuo* to yield the dark red solid as the target compound (**T8**, 0.387 g, 45.0%). m. p. 134–135°C (dichloromethane/methanol, 10:1, R_f_ = 0.4), ^1^H NMR (400 MHz, DMSO-*d*_6_) *δ*: 11.10 (dd, *J* = 8.3, 10.4 Hz, 1H), 7.56–7.36 (m, 1H), 7.32–7.21 (m, 1H), 7.20–7.10 (m, 1H), 7.04 (dt, *J* = 8.9, 8.8 Hz, 1H), 6.97–6.83 (m, 1H), 6.57 (dd, *J* = 8.4, 1.9 Hz, 1H), 4.21–4.00 (m, 2H), 3.60 (s, 4H), 2.55–2.49 (m, 3H), 2.46 (s, 3H), 1.95 (dd, *J* = 8.9, 8.7 Hz, 2H). HRMS (ESI): *m/z* calculated for C_21_H_21_Cl_2_N_3_O_3_ 434.3157, Found 434.3156.

##### 2.1.3.9 5-Methyl-3-[(4-(2-Morpholine-4-yl-Ethoxyl)Phenyl)Imino]-1,3-Dihydro-1*H*-Indole-2-One(T9)

5-Methylisatin (**3c**, 0.319 g, 2.00 mmol, 1.0 equiv) and 1-[2-(4-amino-phenoxyl)ethyl]morpholine (**4a**, 0.444 g, 2.00 mmol, 1.0 equiv) were added to a 50-ml flask, to which was added absolute ethanol (10.0 ml) and acetic acid (1–2 drops) at room temperature under stirring. The reaction mixture was heated to reflux for 10 h until the TLC analysis showed that the reaction was completed. The reaction system was then cooled down to room temperature until there was a small amount of dark red solid being precipitated. A small amount of water was added to the reaction mixture to allow the large amount of solids to be precipitated. The solids were obtained *via* filtration under the reduced pressure. The filter cake was dried under *vacuo* to yield the dark red solids as the desired target compound (**T9**, 0.327 g, 45%). m. p.127∼129°C (dichloromethane/methanol, 10:1, R_f_ = 0.5), ^1^H NMR (400 MHz, DMSO-*d*_6_) *δ*: 10.81 (d, *J* = 8.5 Hz, 1H), 7.26–7.13 (m, 2H), 7.06 (d, *J* = 8.8 Hz, 1H), 6.98 (d, *J* = 8.8 Hz, 1H), 6.90 (d, *J* = 8.9 Hz, 1H), 6.80 (d, *J* = 8.0 Hz, 1H), 6.49 (s, 1H), 4.13 (dt, *J* = 5.0, 5.8 Hz, 2H), 3.65–3.52 (m, 4H), 2.73 (s, 2H), 2.01 (s, 3H). HRMS (ESI): *m/z* calculated for C_21_H_23_N_3_O_3_ 365.4256, Found 365.4258.

##### 2.1.3.10 5-Methyl-3-[(4-(2-Morpholine-4-yl-propoxy)phenyl)imino]-1,3-dihydro-1*H*-indole-2-one(T10)

5-Methylisatin (**3c**, 0.319 g, 2.00 mmol, 1.0 equiv), 1-[2-(2-fluoro-4-aminophenoxyl)propyl] morpholine (**4b**, 0.508 g, 2.00 mmol, 1.0 equiv) were added to a 50-ml flask, to which was added absolute ethanol (10.0 ml) and acetic acid (1–2 drops) at room temperature under stirring. The reaction mixture was heated to reflux for 10 h until the TLC analysis showed that the reaction was completed. The reaction system was then cooled down to room temperature until there was a large amount of orange-yellow solids being precipitated. The solids were obtained *via* filtration under the reduced pressure. The filter cake was dried under *vacuo* to afford the orange-yellow solid as the target compound (**T10**, 0.083 g, 10%). m. p. 117–120°C (dichloromethane/methanol, 10:1, R_f_ = 0.4),^1^H NMR (400 MHz, DMSO-*d*_6_) *δ*: 10.83 (d, *J* = 8.3 Hz, 1H), 7.27 (t, *J* = 8.9 Hz, 1H), 7.20 (d, *J* = 7.9 Hz, 1H), 7.12 (dd, *J* = 9.9, 5.3 Hz, 1H), 7.00 (dd, *J* = 8.1, 1.9 Hz, 1H), 6.81 (d, *J* = 7.8 Hz, 1H), 6.42 (s, 1H), 4.12 (dt, *J* = 6.8, 6.3 Hz, 2H), 3.58 (s, 4H), 2.45 (d, *J* = 6.3 Hz, 2H), 2.31 (t, *J* = 4.7 Hz, 4H), 2.03 (s, 2H), 1.92 (dd, *J* = 3.8, 3.6 Hz, 2H). HRMS (ESI): *m/z* calculated for C_22_H_24_FN_3_O_3_ 397.4427, Found 397.4425.

##### 2.1.3.11 5-Methyl-3-[(4-(3-Morpholine-4-yl-Propoxyphenyl)Imino]-1,3-Dihydro-1*H*-Indole-2-One(T11)

5-Methylisatin (**3c**, 0.319 g, 2.00 mmol, 1.0 equiv), 1-[2-(4-aminophenoxyl)propyl]morpholine (**4c**, 0.470 g, 2.00 mmol, 1.0 equiv) were added to a 50-ml flask, to which was added absolute ethanol (10.0 ml) and acetic acid (1–2 drops) at room temperature under stirring. The reaction mixture was heated to reflux for 10 h until the TLC analysis showed that the reaction was completed. The reaction mixture was then cooled down to room temperature until there was a large amount of orange-red solids being precipitated. The solids were obtained *via* filtration under the reduced pressure. The filter cake was dried under *vacuo* to produce the orange-red solid as the desired target compound (**T11**, 0.476 g, 50%). m. p. 178–180°C (dichloromethane/methanol, 10:1, R_f_ = 0.4), ^1^H NMR (400 MHz, DMSO-*d*_6_) *δ*: 10.80 (d, *J* = 8.2 Hz, 1H), 7.24–7.14 (m, 2H), 7.04 (d, *J* = 8.9 Hz, 1H), 6.97 (d, *J* = 8.8 Hz, 1H), 6.92–6.71 (m, 2H), 6.50 (s, 1H), 4.10–3.98 (m, 2H), 3.61–3.53 (m, 4H), 2.51 (d, *J* = 1.6 Hz, 1H), 2.45 (t, *J* = 7.1 Hz, 2H), 2.38 (s, 4H), 2.28 (s, 1H), 2.01 (s, 3H), 1.95–1.84 (m, 2H). HRMS (ESI): *m/z* calculated for C_22_H_25_N_3_O_3_ 379.4522, Found 379.4526.

##### 2.1.3.12 3-[(3-Chloro-4-(3-Morpholine-4-yl-Propoxy)Phenyl)Imino]-5-Methyl-1,3-Dihydro-1*H*-Indole-2-One(T12)

5-Methylisatin (**3c**, 0.319 g, 2.00 mmol, 1.0 equiv), 1-[2-(2-chloro-4-aminophenoxyl) propylenepropyl]morpholine (**4d**, 0.540 g, 2.00 mmol, 1.0 equiv) were added to a 50-ml flask, to which was added absolute ethanol (10.0 ml) and acetic acid (1–2 drops) at room temperature under stirring. The reaction mixture was heated to reflux for 10 h until the TLC analysis showed the completion of the reaction. The reaction system was then cooled down to room temperature until there was a large amount of orange-yellow solid being precipitated. The solids were obtained *via* filtration under the reduced pressure. The filter cake was dried under *vacuo* to produce the orange-yellow solid as the desired target compound (**T12**, 0.282 g, 34%). m. p. 113–114°C (dichloromethane/methanol, 10:1, R_f_ = 0.4), ^1^H NMR (400 MHz, DMSO-*d*_6_) *δ*: 10.80 (d, *J* = 8.3 Hz, 1H), 7.26–7.12 (m, 1H), 7.05 (d, *J* = 8.6 Hz, 1H), 6.97 (d, *J* = 8.6 Hz, 1H), 6.92–6.71 (m, 2H), 6.50 (s, 1H), 4.10 (dt, *J* = 6.1, 5.8 Hz, 2H), 2.69 (t, *J* = 5.5 Hz, 2H), 2.51 (s, 1H), 2.46 (s, 3H), 2.29 (s, 1H), 2.01 (s, 2H), 1.53–1.47 (m, 4H), 1.39 (d, *J* = 4.7 Hz, 2H). HRMS (ESI): *m/z* calculated for C_22_H_24_ClN_3_O_3_ 413.8973, Found 413.8975.

##### 2.1.3.13 5-Bromo-3-[(4-(2-Morpholine-4-yl-Ethoxyl)Phenyl)Imino]-1,3-Dihydro-1*H*-Indole-2-One(T13)

5-Bromo-isatin (**3d**, 0.449 g, 2.00 mmol, 1.0 equiv), 1-[2-(4-aminophenoxyl)ethyl]morpholine (**4a**, 0.444 g, 2.00 mmol, 1.0 equiv) were added to a 50-ml flask, to which was added absolute ethanol (10.0 ml) and acetic acid (1–2 drops) at room temperature under stirring. The reaction mixture was heated to reflux for 13 h until the TLC analysis showed that the reaction was completed. The reaction system was then cooled down to room temperature until there was a large amount of orange-red solids being precipitated. The solids were obtained *via* filtration under the reduced pressure. The filter cake was dried under *vacuo* to produce the orange-red solid as the target compound (**T13**, 0.610 g, 71%). m. p. 151–153°C (dichloromethane/methanol, 10:1, R_f_ = 0.5), ^1^H NMR (400 MHz, DMSO-*d*_6_) *δ*: 11.06 (d, *J* = 6.4 Hz, 1H), 7.56 (dd, *J* = 8.7, 8.3, 2.0 Hz, 1H), 7.26 (d, *J* = 8.8 Hz, 1H), 7.09 (d, *J* = 8.8 Hz, 1H), 7.01 (d, *J* = 8.8 Hz, 1H), 6.95–6.80 (m, 2H), 6.72 (d, *J* = 1.7 Hz, 1H), 4.20–4.08 (m, 2H), 3.67–3.53 (m, 4H), 2.74 (t, *J* = 5.6 Hz, 2H). HRMS (ESI): *m/z* calculated for C_20_H_20_BrN_3_O_3_ 430.2951, Found 430.2953.

##### 2.1.3.14 5-Bromo-3-[(4(2-Morpholine-4-yl-Propoxy)Phenyl)Imino]-1,3-Dihydro-1*H*-Indole-2-One(T14)

5-Bromo-isatin (**3d**, 0.449 g, 2.00 mmol, 1.0 equiv), 1-[2-(2-fluoro-4-aminophenoxyl)propyl] morpholine (**4b**, 0.508 g, 2.00 mmol, 1.0 equiv) were added to a 50-ml flask, to which was added absolute ethanol (10.0 ml) and acetic acid (1–2 drops) at room temperature under stirring. The reaction mixture was heated to reflux for 9 h until the TLC analysis showed that the reaction was completed. The reaction system was then cooled down to room temperature until there was a large amount of red solids being precipitated. The solids were obtained *via* filtration under the reduced pressure. The filter cake was dried under *vacuo* to yield the red solid as the target compound (**T14**, 0.285 g, 31%). m. p. 143–144°C (dichloromethane/methanol, 10:1, R_f_ = 0.5), ^1^H NMR (400 MHz, DMSO-*d*_6_) *δ*: 11.09 (d, *J* = 8.9 Hz, 1H), 7.68–7.52 (m, 1H), 7.35–7.11 (m, 1H), 7.08–6.96 (m, 1H), 6.91–6.81 (m, 2H), 6.66 (d, *J* = 1.7 Hz, 1H), 4.19–4.05 (m, 2H), 3.58 (t, *J* = 4.4 Hz, 4H), 2.45 (dd, *J* = 2.9, 6.1 Hz, 2H), 2.39 (s, 4H), 1.92 (dd, *J* = 3.2, 6.7 Hz, 2H). HRMS (ESI): *m/z* calculated for C_21_H_21_BrFN_3_O_3_ 462.3121, Found 462.3120.

##### 2.1.3.15 5-Bromo-3-[(4-(3-Morpholine-4-yl-Propoxyphenyl)Imino]-1,3-Dihydro-1*H*-Indole-2-One(T15)

5-Bromo-isatin (**3d**, 0.449 g, 2.00 mmol, 1.0 equiv) and 1-[2-(4-aminophenoxyl)propyl] morpholine (**4c**, 0.470 g, 4.6 mmol, 2.3 equiv) were added to a 50-ml flask, to which was added absolute ethanol (10.0 ml) and acetic acid (1–2 drops) at room temperature under stirring. The reaction mixture was heated to reflux for 13 h until the TLC analysis showed that the reaction was completed. The reaction system was then cooled down to room temperature until there was a large amount of red solids being precipitated. The solids were obtained *via* filtration under the reduced pressure. The filter cake was dried under *vacuo* to yield the red solid as the target compound (**T15**, 0.702 g, 79%). m. p. 181–182°C (dichloromethane/methanol, 10:1, R_f_ = 0.4). ^1^H NMR (400 MHz, DMSO-*d*_6_) *δ*: 11.06 (d, *J* = 8.8 Hz, 1H), 7.55 (dd, *J* = 9.4, 8.3, 1.9 Hz, 1H), 7.27 (d, *J* = 8.8 Hz, 1H), 7.07 (d, *J* = 8.8 Hz, 1H), 7.00 (d, *J* = 8.8 Hz, 1H), 6.93–6.88 (m, 1H), 6.87–6.80 (m, 1H), 6.73 (d, *J* = 1.8 Hz, 1H), 4.10–4.00 (m, 2H), 3.58 (t, *J* = 4.5 Hz, 4H), 2.51 (s, 1H), 2.43 (dd, *J* = 3.4, 6.3 Hz, 2H), 2.37 (s, 4H), 1.96–1.83 (m, 2H). HRMS (ESI): *m/z* calculated for C_21_H_22_BrN_3_O_3_ 444.3217, Found 444.3216.

##### 2.1.3.16 3-[(3-Chloro-4-(3-Morpholine-4-yl-Propoxylpropoxy)Phenyl)Imino]-5-Bromo-1,3-Dihydro-1*H*-Indole-2-One(T16)

5-Bromo-isatin (**3d**, 0.449 g, 2.00 mmol, 1.0 equiv) and 1-[2-(2-chloro-4-aminophenoxyl)propyl] morpholine (**4d**, 0.540 g, 2.00 mmol, 1.0 equiv) were added to a 50-ml flask, to which was added absolute ethanol (10.0 ml) and acetic acid (1–2 drops) at room temperature under stirring. The reaction mixture was heated to reflux for 11 h until the TLC analysis showed that the reaction was completed. The reaction system was then cooled down to room temperature until there was a large amount of red solids being precipitated. The solids were obtained *via* filtration under the reduced pressure. The filter cake was dried under *vacuo* to yield the red solid as the target compound (**T16**, 0.595 g, 62%). m. p. 147–149°C (dichloromethane/methanol, 10:1, R_f_ = 0.4). ^1^H NMR (400 MHz, DMSO-*d*_6_) *δ*: 11.08 (dd, *J* = 8.7, 9.4 Hz, 1H), 7.67–7.50 (m, 1H), 7.32–7.20 (m, 1H), 7.20–7.10 (m, 1H), 7.04 (dt, *J* = 9.0, 8.7 Hz, 2H), 6.94–6.80 (m, 1H), 6.71 (dd, *J* = 7.7, 1.7 Hz, 1H), 4.20–4.01 (m, 2H), 3.57 (d, *J* = 2.5 Hz, 4H), 2.51 (d, *J* = 1.5 Hz, 3H), 2.44 (s, 3H), 2.02–1.80 (m, 2H). HRMS (ESI): *m/z* calculated for C_21_H_21_BrClN_3_O_3_ 478.7667, Found 478.7669.

### 2.2 Biological Activity

#### 2.2.1 Method for Recording the Electrophysiological Data

For cell culture and transfection, Chinese hamster ovary (CHO) cells were cultured in Dulbecco’s modified Eagle’s medium/Ham’s F-12 (DMEM/F-12) medium supplemented with 10% fetal bovine serum and antibiotics (100 IU/ml penicillin and 100 mg/ml streptomycin) at 37°C in a humidified atmosphere with 5% CO_2_ and 95% air. Cells were passaged every 3–4 days with trypsin–EDTA, and a part of treated cells were seeded onto glass cover slips (5 × 3 mm^2^) for later transfection. The mammalian expression vector pcDNA3.1, which contained full-length cDNA encoding human Kv1.5 (hKv1.5), was kindly provided by Professor David Fedida (University of British Columbia, Canada). Wild-type hKv1.5 channel cDNA (0.5 μg) and green fluorescent protein (GFP) cDNA (0.5 μg) were transfected transiently into CHO cells by using Lipofectamine (Invitrogen Life Technologies, Carlsbad, CA, United States ). After transfecting for 48 h, the patch clamp experiments were conducted on GFP-positive cells. To prepare the materials of the drug candidates, the target compounds were dissolved in dimethyl sulphoxide (DMSO) to yield the stock solutions of 50 mM. The concentration of DMSO in the final solution was less than 0.1% (V/V), which had no effects on hKv1.5 channel current in the detective level.

The cells which could radiate the green fluorescence under UV excitation were selected for the experimental data recording. The Kv1.5 current was recorded from the cell which was green fluorescent protein (GFP)-positive 48 h after transfection at room temperature (25°C), using an EPC-8 patch-clamp amplifier (HEKA, Lambrecht, Germany), which has been recorded in the reported literature (Wu et al., 2009). Patch electrodes were fabricated from the glass capillaries (Narishige Scientific Instrument Lab, Tokyo, Japan) using a P-97 horizontal puller (Sutter Instrument Co., Novato, CA, United States ). These electrodes had a resistance of 2.0–3.0 MΩ when they were filled with the pipette solution containing 70 mM potassium aspartate, 40 mM KCl, 10 mM KH_2_PO_4_, 1 mM MgSO_4_, 3 mM adenosine 5-triphosphate (disodium salt; Sigma Chemical Company, St. Louis, MO, United States ), 0.1 mM dilithium salt of guanosine 5′-triphosphate (Roche Diagnostics GmbH Company, Mannheim, Germany), 5 mM EGTA, and 5 mM HEPES (pH was adjusted to 7.2 with 1 M KOH solution). Cells attached to glass cover slips were transferred to a recording chamber (0.5 ml in volume) mounted on the stage of an inverted microscope (ECLIPSE TE2000-U, Nikon, Tokyo, Japan), and perfused continuously at a flow rate of 1–2 ml/min with Tyrode solution containing 140 mM NaCl, 5.4 mM KCl, 1.8 mM CaCl_2_, 0.5 mM MgCl_2_, 0.33 mM NaH_2_PO_4_, 5.5 mM glucose, and 5.0 mM HEPES (pH was adjusted to 7.4 with 1 M NaOH). The data were then low-pass filtered at 10 kHz and sampled at 50 kHz during measuring the activation time course of hKv1.5 current, and the series resistances were usually compensated by 80%.

The hKv1.5 channel current was elicited by applying 300 ms depolarizing steps from a holding potential of −80 mV to various levels of −50 mV to +50 mV in 10 mV steps with a return potential of −40 mV. The stimulus interval of each stepwise was longer than 10 s to ensure that the channel fully recovered from the inactive state except for the frequency-dependent experiment. Apart from being specified, 30 mV was used to depolarize the voltage when the inhibition effects of drugs on the current were detected while using 300-ms bandwidth to excite the final current. The voltage-dependent activation of the hKv1.5 channel was assessed by fitting with a Boltzmann equation:

$$I_{\text{tail}}=\;1\text{/}\left(1\;+\text{ exp}\left(\left(V_{1\text{/}2}-V\text{m}\right)\text{/k}\right)\right)$$ (1)

In the above equation, *I*_tail_ is the tail current amplitude normalized with reference to the maximum value (as the 100%) measured at +50 mV; *V*_1/2_ is the voltage at half-maximal activation; *V*_m_ is the test potential and *k* is the slope factor.

The inhibition rates (IRs) of the target compounds against the Kv1.5 channel were calculated based on the value of *I*_tail,_ which were recorded before and after the drug administration. The equation for calculating the IR was shown as follows.

$$\text{IR}=I_{\text{tail After the drug administration}}\text{/}I_{\text{tail Before the drug administration}}$$ (2)

The concentration-response curve for inhibition of hKv1.5 current by the control group was drawn by a least-squares fit of a Hill equation: %Control = 1/(1 + (IC_50_/[D])^nH^), where %Control represents the current in the presence of the drug normalized with reference to the control amplitude (expressed as a percentage). IC_50_ is the concentration of the control group causing a half-maximal inhibition. n_H_ is the Hill coefficient and [D] is drug concentration. The apparent rate constants for binding (*k*_+1_) and unbinding (*k*_-1_) were obtained from fitting the equation: t_D_ = 1/(*k*_+1_ [D] + *k*_-1_), where t_D_ is the time constant induced by the drug, which was calculated from a single exponential fit to the traces of current decay during the depolarizing step to +30 mV. The apparent dissociation constant *K*_D_ is expressed as *K*_D_ = *k*_-1_/*k*_+1_. The deactivation kinetics was determined by fitting a single exponential function to the tail current trace.

All of the averaged data are presented as mean ± SEM (*n* = number of cells). Student’s *t*-test or ANOVA with Dunnett’s *post hoc* test was used to evaluate the statistical significance, which is considered to be statistically significant if a *p* value of < 0.05 was obtained.

#### 2.2.2 Method for Recording the Vasodilation Activity

Besides the Kv1.5 inhibition activity, the vasodilation activity of the Kv1.5 inhibitors was also tested *in vitro* based on our previous research work (Chen et al., 2016a; Chen et al., 2016b; Chen et al., 2016c; Chen et al., 2017; Chen et al., 2018). Briefly, the vasoconstriction model of the rat superior mesenteric artery was induced by 60 mM KCl or 10 μM PE, and target compounds were added to detect the vasodilation effects on pre-contracted vessels by using the microvascular tension measurement system Myograph (DMT620M, Danish Myo Technology A/S Company, Denmark).

The male Sprague-Dawley (SD) rats (SPF, 8 weeks old, 200–250 g) were obtained as the experimental animals from the Animal Centre of Xi’an Jiaotong University. The SD rats were euthanized using CO_2_. The superior mesenteric artery was gently removed and freed from adhering tissue under a dissecting microscope and then cut into 2- to 3-mm cylindrical arterial rings. The arterial rings were immersed in eight individual myograph baths (37°C) (Organ Bath Model 700MO, J.P. Trading, Aarhus, Denmark) containing 5 ml physiologic buffer solution (PSS) (pH 7.4, 119 mM NaCl, 4.6 mM KCl, 15 mM NaHCO_3_, 1.2 mM NaH_2_PO_4_, 1.2 mM MgCl_2_, 1.5 mM CaCl_2_, and 5.5 mM glucose). The myograph baths were continuously aerated with 5% CO_2_ in O_2_.

The arterial segments were mounted for continuous recording of isometric tension using LabChart7 Pro software (AD Instruments, Hastings, United Kingdom). A resting tension of 2 mN was applied to each arterial ring, which was stabilized under this tension for at least 1.5–2.0 h. Then the rings were exposed to a potassium buffer solution (KPSS) (60 mM KCl in PSS). The maximum contraction induced by potassium was considered as a reference for contractile capacity. When potassium-induced reproducible responses were over 5 mN, the arterial rings could be used for experiments.

The vascular relaxation effects after the cumulative administration of tested compounds on artery rings was analyzed by the concentration-response curve for the chemicals (10^–10^–10^−4^ M) after initial potassium-induced contraction. DMSO was chosen as the control. The following formula is used to calculate the vasodilation rate of the target compound against the pre-contracted vascular rings (Equation 3).

$$\begin{array}{l} \text{Vasodilation rate }=\;\left({\text{Isometric tension value after K}}^{+}\text{ or PE preconstruction }-\text{ Isometric tension value after target compound dosing}\right)\\ \text{ /}\left({\text{Isometric tension value after K}}^{+}\text{ or PE preconstruction }-\text{ Baseline tension value}\right)\text{× }100\text{\% } \end{array}$$ (3)

Equation 3. Calculation equation of the vasodilation rate of the tested compounds.

The studies *in vitro* were approved by the Laboratory Animal Ethics Administration Committee of Xi’an Medical University and implemented according to the Guide for the Care and Use of Laboratory Animals Published by the United States National Institutes of Health (NIH Publication No. 85–23, revision in 1996).

All data were expressed as mean ± SD (standard deviation). The tested compound-induced vasodilation was presented as a percentage of response induced by KCl. One-way ANOVA with Dunnett’s post-test was applied for comparisons of more than two data sets. A *t*-test was applied for comparisons of two data sets. A *p* value of < 0.05 was considered to be significant. The analysis was performed by using GraphPad software (GraphPad Software, San Diego, United States ).

### 2.3 Molecular Docking

#### 2.3.1 Calculation and Preparation of the Molecules

The chemical structures of all the target molecules were drawn using ChemBio Draw Ultra 12.0, which were opened in ChemBio3D Ultra 12.0. These molecules were named and minimized *via* the MM-semi-empirical method, which were then saved as Mol2 files one by one. The molecular descriptors were also calculated using ChemBio3D Ultra 12.0, which include the parameters of Stretch, Bend, Stretch-Bend, Torsion, Non-1,4 VDW, 1,4 VDW, Dipole/Dipole, and Total Energy.

The Mol2 file of each molecule was opened in the Sybyl workstation and was renamed again with the same name. A new database including all these 16 compounds was established based on the above information. All of the molecules were minimized in a Molecule Spread Sheet (MSS) file using the default value except for the following four settings. The force field was chosen as “Tripos.” The charge was chosen as “Gasteiger-Huckel.” “Max. Iterations” was set to be 10,000. “Gradient” was set to be 0.005. “Color option” was set to be Force. The minimization was repeated while there is still “Some columns may need to be recomputed” popped up in the column of “Console Command” of the Sybyl work-space. Minimization could also be stopped until the total energy values obtained from the last three conformation states of the molecule were the same without changing again.

#### 2.3.2. Molecular Docking of the Compounds With the Target Kv1.5

Molecular modeling was carried out using the Tripos molecular modeling packages Sybyl-X2.0. All the molecules for docking were built using standard bond lengths and angles from Sybyl-X2.0/base Builder and were then optimized using the Tripos force field for 2000 generations two times or more, until the minimized conformers of the ligand were the same. The flexible docking method, called Surflex-Dock, docks the molecules automatically into the binding site of the receptor by using a protocol-based approach and an empirically derived scoring function. The protocol is a computational representation of a putative ligand that binds to the intended binding site and is a unique and essential element of the docking algorithm. The scoring function in Surflex-Dock, which contains hydrophobic, polar, repulsive, entropic, and salvation terms, was trained to estimate the dissociation constant (Kd) expressed in −log (Kd)^2^.

The 3D structure data of Kv1.5 was derived from its Kv1.2 homologues protein *via* the molecular homology method. Surflex-Dock default settings were used for other parameters, such as the number of starting conformations per molecule (set to 0), the size to expand the search grid (set to 8 Å), the maximum number of rotatable bonds per molecule (set to 100), and the maximum number of poses per ligand (set to 20). During the docking procedure, all of the single bonds in side chains of residues inside the defined binding pocket were regarded as rotatable or flexible. The ligands were allowed to rotate on all single bonds and move flexibly within the tentative binding pocket. The atomic charges were recalculated using the Kollman all-atom approach for the protein and the Gasteiger Hückel approach for the ligand. The binding interaction energy was calculated to include van der Waals, electrostatic, and torsional energy terms defined in the Tripos force field.

## 3 Results and Discussion

### 3.1 Rational Drug Design

#### 3.1.1 Molecular Skeleton Design

Based on target–ligand interaction and structure-activity analysis of traditional inhibitors and its simulated 3D structure of target Kv1.5, a series of compounds (T1–T16), containing both morpholine and 1*H*-indole linked with a chain-like structure (-N(CH_2_)_n_O-) in one molecule, were designed to enhance therapeutic effects of the anti-AF drugs (Figure 1).

#### 3.1.2 Molecular Skeleton Docking Analysis

##### 3.1.2.1 Building of Simulated 3D Structure of Kv1.5

**FIGURE 2:**
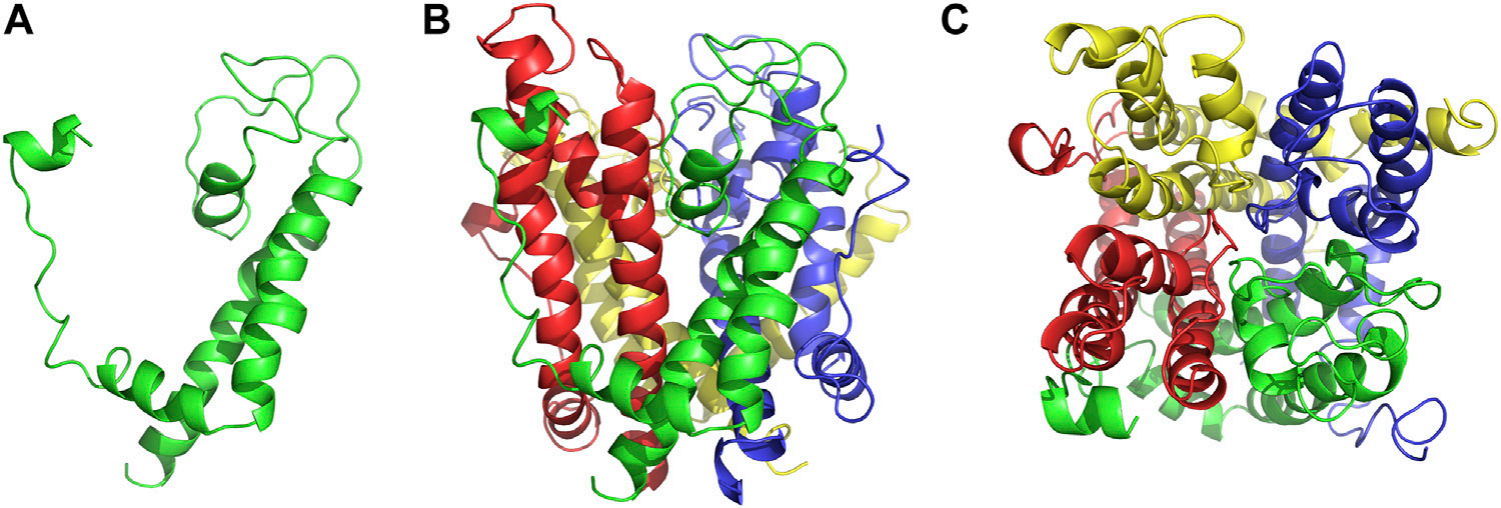
Simulated 3D structure of the transmembrane region of the Kv1.5 channel. **(A)** A single subunit of the transmembrane region of Kv1.5 viewed from the side. **(B)** Four subunits of the transmembrane region of Kv1.5 viewed from the side. **(C)** Four subunits of the transmembrane region of Kv1.5 viewed from the extracellular side of the pore. The subunits are represented as ribbons and colored individually.

As an anti-AF drug target with high selectivity on the atrial muscle cells, the undetermined crystal structure of the Kv1.5 channel impeded further new drug development. Herein, the simulated 3D structure of the Kv1.5 channel as the drug target was built based on the crystal structure of its homolog Kv1.2 channel and its amino acid sequence using the homology modeling technique. The crystal structure of the Kv1.2 channel (PDB ID: 2A79) was successfully determined by X-ray diffraction several years ago (Long et al., 2005). To be specific, the complete Kv1.2 channel consisted of the same four subunits and thereby formed a large tetramer. One subunit of Kv1.2 included the N terminus forming a T1 domain at the intracellular membrane surface, six transmembrane segments (S1–S6), and the C terminus. The transmembrane pore was composed by S5, pore helix, selectivity filter, and S6. Besides, the S1–S4 segments formed the voltage sensor (Long et al., 2005). The simulated 3D structure of the transmembrane region of the Kv1.5 channel was built using the homology modeling technique based on the transmembrane region of the Kv1.2 channel (PDB ID: 2A79), which reached over 70% amino acid sequence homology with the Kv1.5 channel. Unsurprisingly, the transmembrane region of Kv1.5 was similar to that of Kv1.2 and the single subunit of Kv1.5 mainly contained the pore mentioned above and the S4–S5 linker helix (Figure 2A). Furthermore, the four subunits of Kv1.5 formed a bundle crossing with a wide top and a narrow bottom; thus, the whole channel appeared like an inverted cone (Figures 2B,C).

##### 3.1.2.2 Parameter Results

The multiple descriptors of the target compounds *via* various kinds of software are summarized in Table 1 and Table 2, respectively.

**TABLE 1:** Molecular descriptors of the target compounds calculated *via* ChemBio 3D software.

No	Molecular weight (kcal/mol)	Stretch (kcal/mol)	Bend (kcal/mol)	Stretch- bend (kcal/mol)	Torsion (kcal/mol)	Non-1,4 VDW (kcal/mol)	1,4 VDW (kcal/mol)	Dipole/withDipole (kcal/mol)	Total energy (kcal/mol)
T1	369.3895	1.7644	25.2043	0.4045	−5.8843	−1.9487	24.9111	−2.8714	41.5799
T2	401.4065	1.9527	27.1264	0.4667	−10.0194	−0.7371	25.2394	−1.9294	42.0992
T3	383.4161	1.9183	27.0527	0.4813	−10.0192	−0.7051	25.2687	−2.9527	41.0441
T4	417.8611	2.1221	28.0927	0.5593	−9.0782	−0.4922	25.8207	−1.5999	45.4245
T5	385.8441	1.8749	26.8363	0.4798	−10.0375	−0.7179	25.2327	−2.9851	40.6833
T6	417.8611	1.9882	26.5327	0.5054	−4.5569	−1.8366	25.7237	−1.6266	46.7299
T7	399.8707	1.8651	25.3230	0.4472	−5.8593	−2.2297	26.1172	−2.9479	42.7157
T8	434.3157	2.1495	28.0487	0.5790	−9.0782	−0.6392	26.4072	−1.6499	45.8171
T9	365.4256	2.0521	28.0600	0.5320	−9.3115	−0.3376	25.0976	−3.1901	42.9024
T10	397.4427	2.1532	28.3585	0.5444	−9.2980	−0.4900	25.6598	−2.2980	44.6299
T11	379.4522	1.9504	25.4683	0.4308	−6.5666	−2.2587	26.2950	−3.6802	41.6390
T12	413.8973	2.2318	28.2231	0.5625	−9.7988	−0.6820	26.5886	−2.4528	44.6724
T13	430.2951	1.9768	27.9482	0.5642	−8.5920	−0.3444	25.0620	−2.5829	44.0318
T14	462.3121	1.9122	25.4598	0.4426	−5.9980	−2.3330	26.2287	−2.1015	43.6109
T15	444.3217	2.0521	28.1294	0.5826	−8.5762	−0.5119	25.6865	−2.4850	44.8776
T16	478.7667	2.1624	28.0796	0.5904	−9.0770	−0.7066	26.5679	−1.6945	45.9222

**TABLE 2:** Various energy data of the target compounds from energy minimization *via* Sybyl.

Compounds	Bond stretching energy	Angle bending energy	Torsional energy	Out of plane bending energy	1–4 van der Waals energy	van der Waals energy	1–4 electrostatic energy	Electrostatic energy	Total energy (kcal/mol)
T1	0.475	18.409	5.011	0.006	−0.827	−3.767	0.66	−0.953	19.015
T2	0.536	18.564	4.932	0.005	−0.726	−3.877	1.196	−0.353	20.278
T3	0.522	18.494	5.019	0.006	−0.625	−3.871	−0.838	−0.272	18.436
T4	0.514	19.087	5.61	0.005	−1.248	−4.622	0.173	−0.107	19.413
T5	0.477	18.794	2.56	0.007	−1.089	−3.366	1.259	−0.138	18.505
T6	0.599	20.184	2.832	0.005	−0.989	−3.013	1.455	−0.515	20.557
T7	0.521	18.49	5.035	0.006	−0.781	−3.928	−0.389	0.159	19.113
T8	0.514	19.086	5.615	0.005	−1.404	−4.673	0.621	0.308	20.073
T9	0.522	20.491	3.579	0.006	−1.531	−3.377	−0.116	0.176	19.75
T10	0.532	19.224	5.747	0.005	−1.331	−4.409	0.518	0.732	21.017
T11	0.522	18.512	5.401	0.006	−0.871	−4.166	−1.486	0.749	18.667
T12	0.516	19.109	5.978	0.005	−1.494	−4.91	−0.476	0.89	19.617
T13	0.471	19.421	3.047	0.007	−1.49	−3.632	1.315	0.026	19.165
T14	0.534	18.566	4.957	0.005	−0.939	−3.97	1.732	0.2	21.086
T15	0.516	19.178	5.425	0.006	−1.22	−4.139	−0.333	0.512	19.945
T16	0.514	19.089	5.616	0.005	−1.461	−4.704	0.712	0.439	20.21

##### 3.1.2.3 Molecular Skeleton Docking Analysis

The simulated 3D structure of the Kv1.5 channel was used for docking analysis with all 16 target compounds. The docking result with the total scores (TSs) of each compound with the target protein Kv1.5 are summarized in Table 3.

**TABLE 3:** Docking scores of the target compounds with Kv1.5 *via* Sybyl surflex software.

Compounds	Crash	Polar	D_Sore	PMF_Score	G_Score	Chem score	CScore	Global CScore	TScore
T1	−2.2288	0.0009	−124.122	6.808	−218.083	−13.9141	3	2	4.4915
T2	−3.7555	0.5788	−128.67	15.2886	−239.458	−15.666	3	2	4.0852
T3	−1.2455	0.0003	−123.575	0.3312	−207.486	−14.8693	2	2	5.4447
T4	−3.2448	0	−130.993	5.0454	−251.84	−15.4181	3	3	4.2298
T5	−3.6345	0.0967	−127.816	−6.5237	−233.514	−19.1399	5	3	3.0305
T6	−2.7651	0.2745	−135.296	−4.7169	−233.066	−18.451	2	3	3.358
T7	−2.3765	0	−128.67	4.8988	−238.388	−16.0943	2	2	4.8958
T8	−3.3895	0.0128	−136.89	0.9895	−241.838	−17.7217	2	4	3.2077
T9	−3.5624	0.0004	−127.977	16.4223	−270.607	−16.2803	3	2	4.0118
T10	−3.7264	0.6306	−131.849	25.8995	−239.764	−16.0418	1	2	4.0764
T11	−3.2851	0.1043	−136.128	17.9942	−265.404	−17.4443	3	3	5.4632
T12	−4.0651	0	−136.509	2.098	−265.734	−16.8519	4	4	3.461
T13	−2.2642	0	−125.186	−5.4639	−212.28	−15.7779	2	2	3.7695
T14	−3.6425	0.2116	−135.866	−0.1636	−248.562	−19.0016	3	4	2.3214
T15	−2.0243	0	−133.927	2.671	−236.825	−16.8604	3	3	5.4031
T16	−2.9108	0.0036	−135.657	4.4222	−247.875	−17.9901	2	4	3.4446

There are six compounds with CScore >3 and TScore >4. The docking TScore of **T11** > **T15** > **T1** > **T4** > **T2** > **T9** are 5.4632, 5.4031, 4.4915, 4.2298, 4.0852, and 4.0118, respectively.

Eight amino acid residues including T479, T480, R487, A501, I502, I508, V512, and V516 were identified to constitute the potential binding sites for some Kv1.5 channel blockers, such as vernakalant (Eldstrom et al., 2007), bupivacaine (Franqueza et al., 1997), anthranilic-acid derivative S0100176 (Decher et al., 2004), and arachidonic acid (Bai et al., 2015). Based on the above analysis, molecular docking of all the target compounds with the Kv1.5 channel was conducted *via* the Surflex dock module on the Sybyl workstation. According to the docking scores in Table 3, we choose two typical compounds (T11, CScore = 3, TScore = 5.4632; T10, CScore = 1, TScore = 4.0764) for analyzing the docking results.

**FIGURE 3:**
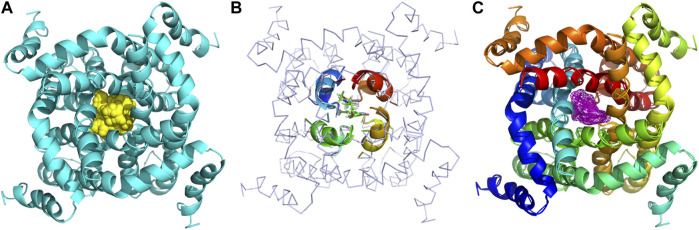
Simulated 3D complex structure of compound **T11** with its target Kv1.5 channel using Surflex Docking. **(A)** Kv1.5 channel was shown as a tetramer colored in cyan, and the binding site in the center was shown in the surface colored in yellow. **(B)** Binding site of the Kv1.5 channel was shown in the rainbow cartoon. Compound **T11** was shown in the green and white stick situated in the center of the channel site. **(C)** Kv1.5 channel was shown in the rainbow cartoon. Compound **T11** in the center of the Kv1.5 channel site was shown in the magenta mesh.

**FIGURE 4:**
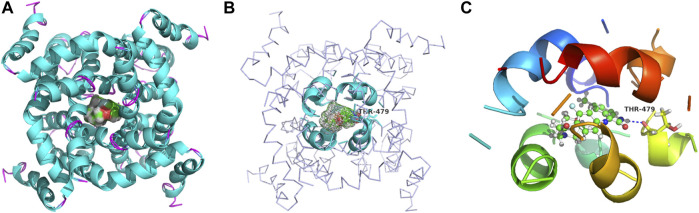
Simulated 3D complex structure of compound **T10** with its target Kv1.5 channel using Surflex Docking. **(A) T10** was shown in the surface while Kv1.5 was shown in the cyan cartoon. **(B)** Binding site around compound **T10** (shown in mesh) was shown in the cyan cartoon. **(C)** Hydrogen bond (blue line) was formed between 1*H* of indole and O of the carbonyl group on the backbone of Thr479 amino acid residue.

The binding models of two compounds indicated that the binding site of the compounds were all in the central cavity of the Kv1.5 channel (Figure 3, Figure 4). The binding manner of **T10** and **T11** is similar (Figure 3, Figure 4). For both compound **T11** and compound **T10**, hydrogen on 1*H*-indole ring is close to T480 on C chain (Figure 3), which forms a hydrogen bond with oxygen of the carbonyl group on the backbone of T479 on C chain (Figure 4).

### 3.2 Chemistry

**FIGURE 5:**
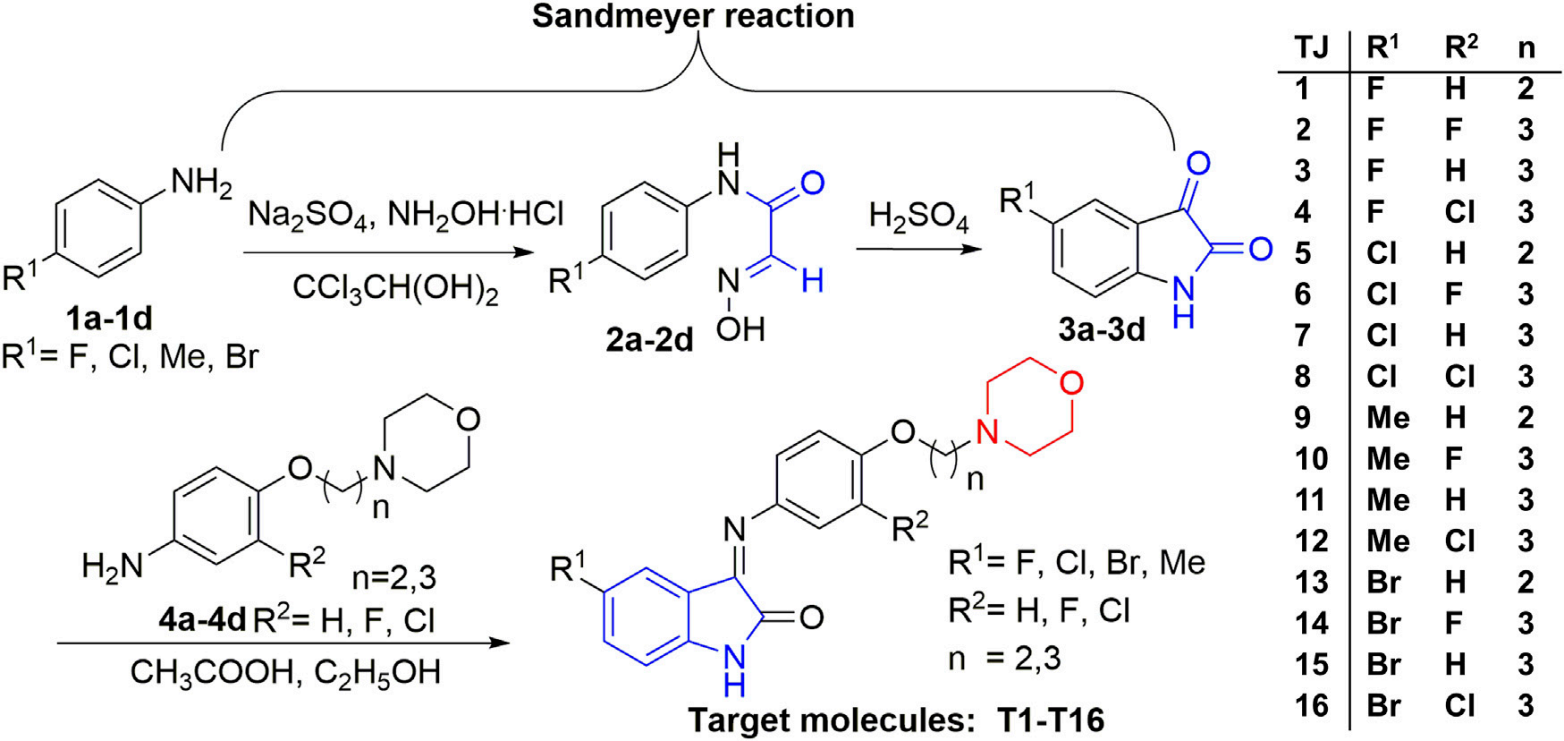
Synthesis route of the target compounds.

The synthesis route of target compounds with the structure of 3-(morpholine substituted aromatic imine)-1*H*-indole is shown in Figure 5. The whole synthesis route contained three main reaction steps starting from the raw material of substituted aromatic amines (**1a-1d**). The important intermediates are 5-substituted isatin derivatives (**3a-3d**). Some of the main materials (**4a-4d**) were synthesized in the pharmaceutical chemistry laboratory of Xi’an Jiaotong University from the commercially available materials, including various substituted aromatic amines and substituted morpholines (Figure 5).

The yields and properties of the important intermediates including 4-substituted-*N*-hydroxyloxime acetyl aniline (**2a-2d**) and 5-substituted isatin (**3a-3d**) are summarized in Supplementary Table S1.

### 3.3 Biological Activity

#### 3.3.1 The Biological Analysis of Kv1.5 Inhibition

The Kv1.5 channel inhibition effects of all the target compounds were tested with the patch clamp technique. The results demonstrated that some compounds showed certain inhibition effects against the Kv1.5 channel among sixteen target compounds. Compound **T16** showed the highest activity at the concentration of 100 μM with the inhibition rate of 70.8%. Compound **T5** showed the relative higher activity at the concentration of 100 μM than the rest of the compounds with the inhibition rate of 57.5%. The sequence of the biological activity is listed as follows: **T16** > **T5** > **T9** > **T10** > **T11** > **T7**. Compounds **T9**, **T10**, **T11**, and **T7** showed the certain inhibitory effects at the low concentration (50 μM); the inhibition rates of these compounds are 20.8, 15.0, 11.3, and 9.5%, respectively (Table 4). Obviously, compounds T16 and T5 exhibited favorable inhibition effects against Kv1.5, which would provide promising lead compounds for anti-AF drug development.

**TABLE 4:** Structure and the inhibitory actions on Kv1.5 of the target compounds.

Compounds	Structure	The concentration of the mother liquid (50 mM)	Molecular weight	Inhibition rate (concentration of the sample)
T1	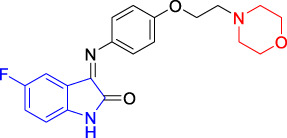	18.47 mg/ml	369.3895	
T2	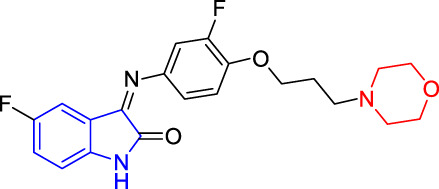	20.07 mg/ml	401.4065	
T3	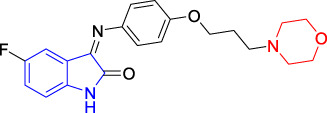	19.17 mg/ml	383.4161	
T4	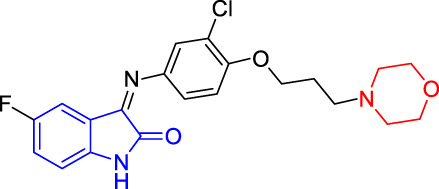	20.89 mg/ml	417.8611	
T5	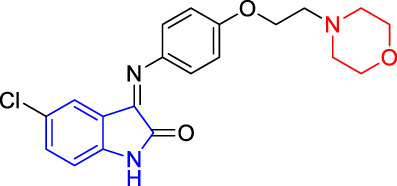		385.8441	57.5% (100 μM)
T6	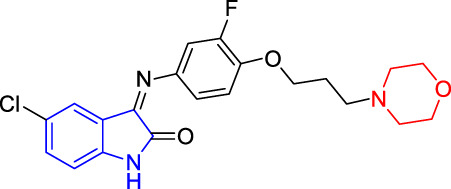	20.89 mg/ml	417.8611	
T7	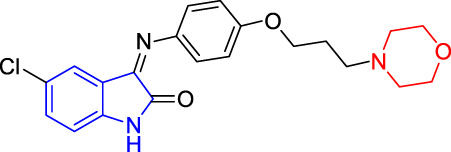		399.8707	9.5% (50 μM)
T8	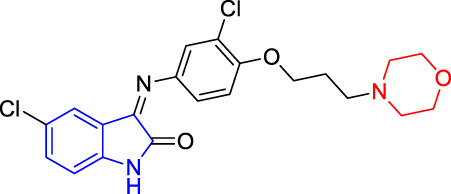	21.76 mg/ml	434.3157	
T9	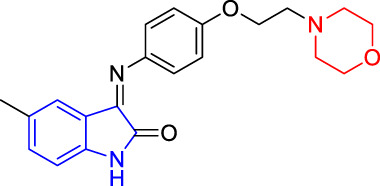	18.27 mg/ml	365.4256	20.8% (50 μM)
T10	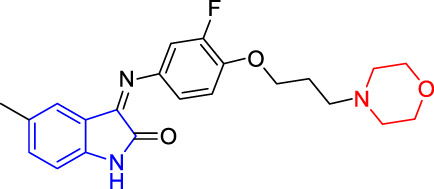	19.87 mg/ml	397.4427	15% (50 μM)
T11	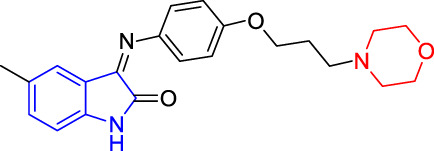	18.97 mg/ml	379.4522	11.3% (50 μM)
T12	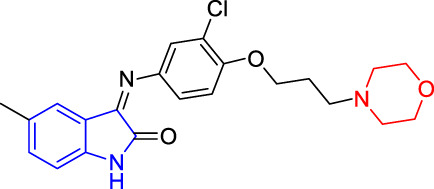	20.695 mg/ml	413.8973	
T13	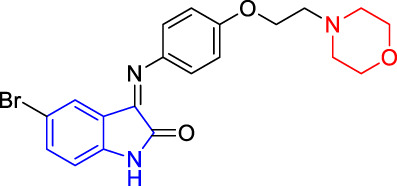	21.514 mg/ml	430.2951	
T14	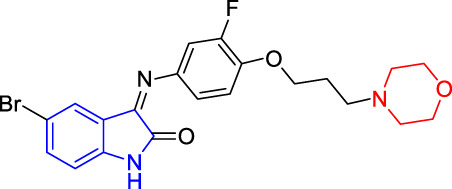	23.12 mg/ml	462.3121	
T15	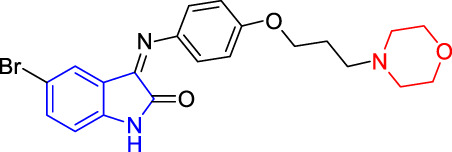	39.5 mg/ml	444.3217	0% (100 μM)
T16	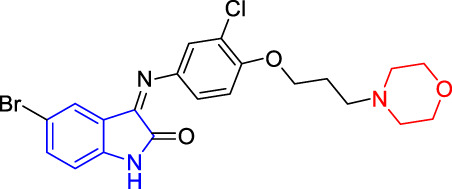	37.3 mg/ml	478.7667	70.8% (100 μM)
LY294002 (control sample)	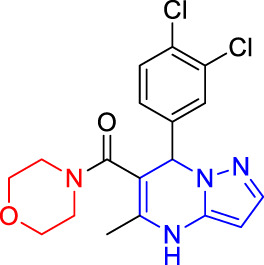	IC_50_ = 7.9 μM		100% (50 μM)

#### 3.3.2 The Biological Analysis of Vasodilation Activity

**FIGURE 6:**
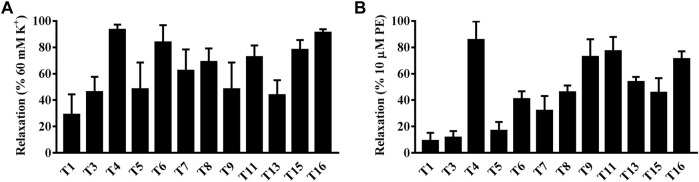
Vasodilation effects of target compounds on superior mesenteric artery rings *in vitro* pre-contracted by KCl (60 mM) or by phenylephrine hydrochloride (PE, 10 μM). **(A)** KCl. **(B)** PE.

There were twelve compounds (100 μM) among sixteen ones showed obvious vasodilation effects (28.96–93.39%) on superior mesenteric artery rings of SD rats *in vitro* pre-contracted by KCl (60 mM). To be specific, their vasodilation rates were as follows: 93.39% (**T4**), 91.11% (**T16**), 83.88% (**T6**), 78.12% (**T15**), 72.59% (**T11**), 68.92% (**T8**), 62.26% (**T7**), 48.30% (**T5** and **T9**), 46.06% (**T3**), 43.78% (**T13**), and 28.96% (**T1**). Obviously, compounds **T4** (93.39%) and **T16** (91.11%) showed extraordinary vasodilation rates compared to other compounds (Figure 6A).

There were twelve compounds (100 μM) among sixteen that showed the obvious vasodilation effects (9.12–85.63%) on the superior mesenteric artery rings of SD rats *in vitro* pre-contracted by PE (10 μM). To be specific, their relaxation rates were as follows: 85.63% (**T4**), 77.23% (**T11**), 72.75% (**T9**), 69.33% (**T16**), 53.88% (**T13**), 45.91% (**T8**), 45.62% (**T15**), 40.68% (**T6**), 31.96% (**T7**), 16.68% (**T5**), 11.65% (**T3**), and 9.12% (**T1**), respectively. Obviously, four samples including **T4** (85.63%), **T11** (77.23%), **T9** (72.75%), and **T16** (69.33%) showed the extraordinary relaxation rates compared to other compounds (Figure 6B).

**FIGURE 7:**
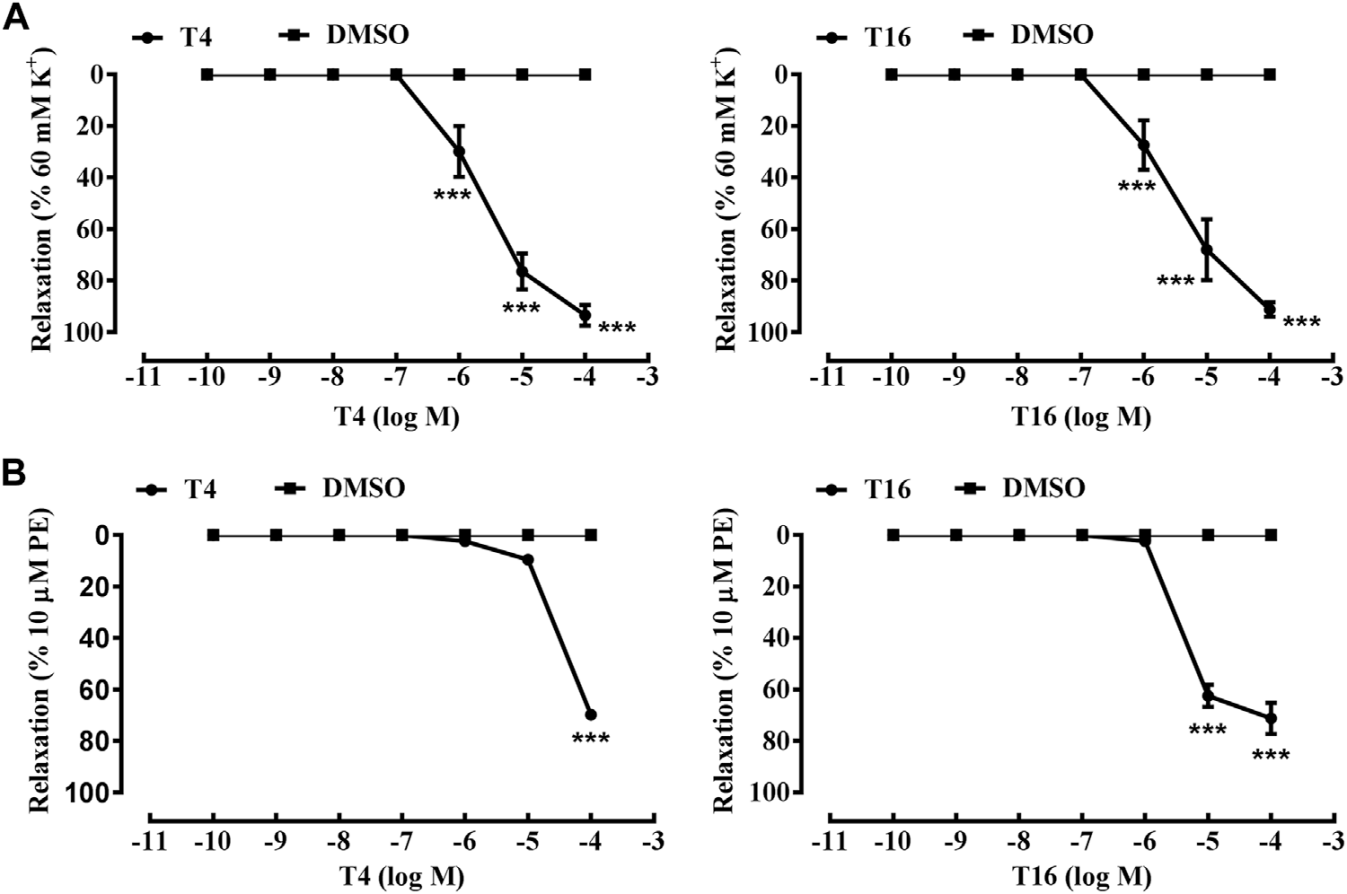
Vasodilation effects of compounds **T4** and **T16** on superior mesenteric artery rings *in vitro* pre-contracted by KCl (60 mM) or by phenylephrine hydrochloride (PE, 10 μM). **(A)** KCl. **(B)** PE. ***p < 0.05 versus a DMSO control (*n* = 3) indicates a significant difference compared with the control (DMSO).

Interestingly, both compounds **T4** and **T16** showed an outstanding vasodilation response on artery rings *in vitro* pre-contracted by KCl in a concentration-dependent manner. The vasodilation effects of compound **T4** started at the concentration of 10^−7^ M, which could rise to 90.70% at the concentration of 10^−4^ M. Similarly, under the same level of concentration, the vasodilation response of compound **T16** (10^−4^ M) was up to the highest value of 94.12% (Figure 7A). Both compounds **T4** and **T16** showed the remarkable vasodilation response on artery rings *in vitro* pre-contracted by PE in a concentration-dependent manner, which took effects at the concentration of 10^−7^ M. The vasodilation effects of compound **T4** (10^−4^ M) could be up to 72.20%; compound **T16** (10^−4^ M) was 61.00% (Figure 7B).

In short, among sixteen target compounds, twelve compounds displayed desirable vasodilation effects on the superior mesenteric artery of SD rats *in vitro* pre-contracted by KCl or PE. As outstanding representatives, compounds **T4** and **T16** exhibited much more remarkable vasodilation effects than other compounds, which would provide reliable lead compounds for anti-hypertension drug development.

### 3.4 Molecular Interaction Analysis of Compound T16 With Its Target Kv1.5

For both Kv1.5 channel inhibitory activity and vasodilation activity, T16 was the optimal one. As a result, T16 was analyzed with regard to its molecular interaction with its target Kv1.5 with the molecular docking.

**FIGURE 8:**
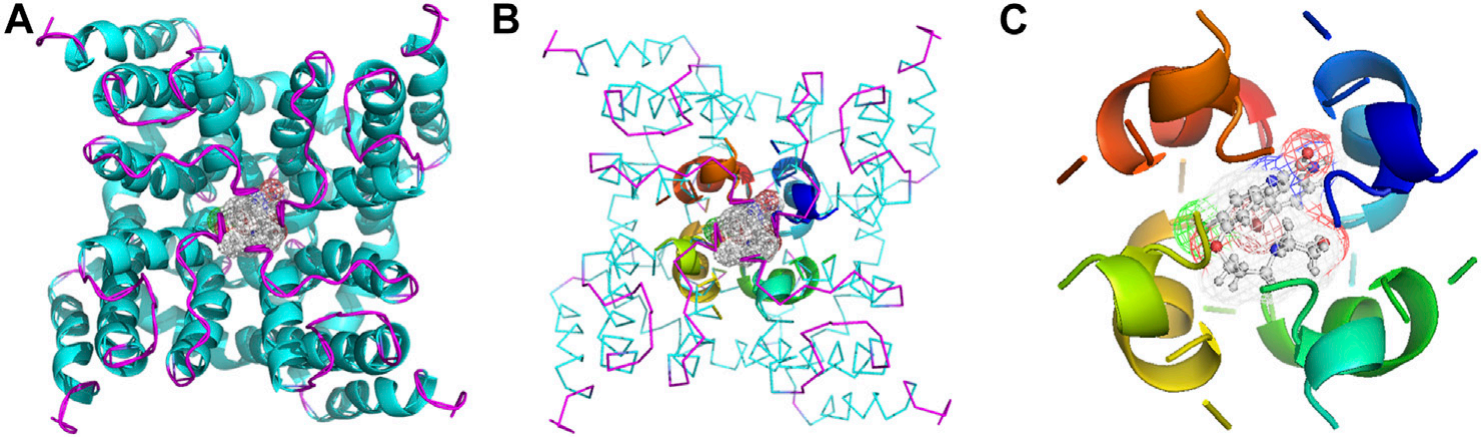
Simulated 3D complex structure of compound **T16** with its target Kv1.5 channel using Surflex Docking. **(A)** Kv1.5 channel was shown according to its secondary structure, in which the α-helixes were shown in cyan, and β-sheets were shown in magenta. Compound T16 situated in the center of the channel was shown in mesh. **(B)** Binding site was shown in the rainbow cartoon. Compound **T16** situated in the center of the channel was shown in mesh. Kv1.5 channel was shown in ribbon, the color of which was shown according to the secondary structure. **(C)** Binding site of the Kv1.5 channel was shown in the rainbow cartoon. Compound **T16** in the center of the binding site was shown in the ball and stick model, which was surrounded with electronic mesh.

**FIGURE 9:**
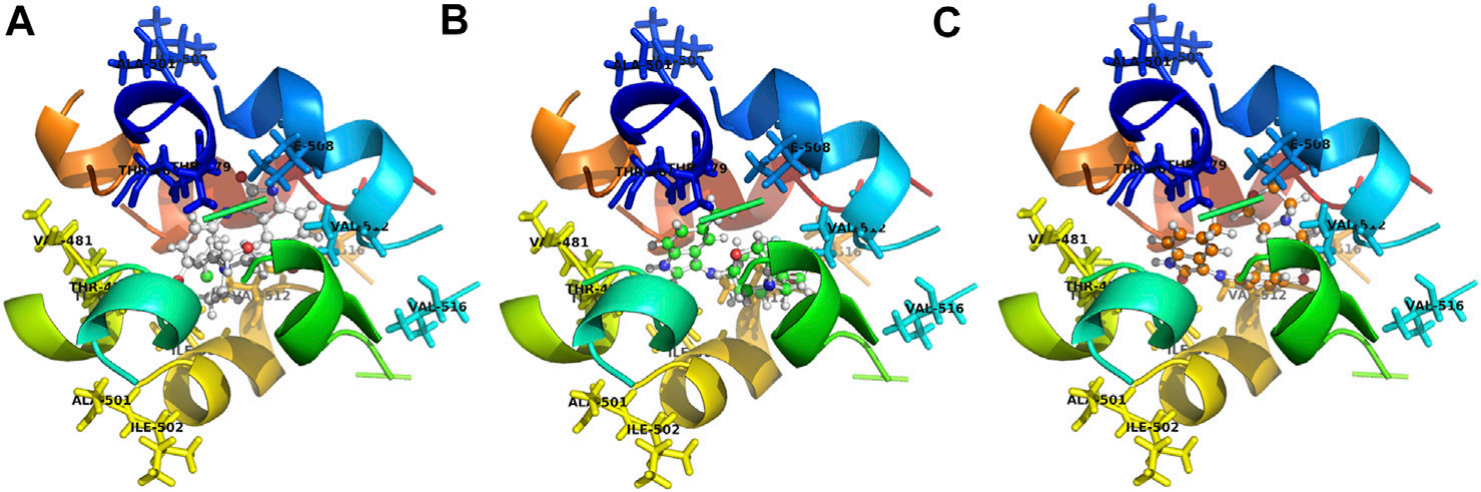
Contrasting simulated molecular interaction models of compounds **T16, T11**, **T10** and their target Kv1.5 channel. The binding site of Kv1.5 channel is composed of the following amino acid residues: T479, T480, A501, I502, I508, V512, and V516 in the S6 domain and selectivity filter of a single subunit of the channel. The binding model of compounds **T11** and **T10** is similar, which is different from compound **T16**. **(A)** compound **T16** (C: gray, H: gray, O: red, N: blue); **(B)** compound **T11** (C: green, H: gray, O: red, N: blue); **(C)** compound **T10** (C: orange, H: gray, O: red, N: blue). A chain was shown in blue, B chain was shown in green, C chain was shown in yellow, and D chain was shown in red.

The binding models of three compounds (**T16, T11**, and **T10**) indicated that the binding site of the compounds were all in the central cavity of the Kv1.5 channel (Figure 3, Figure 4, and Figure 8, Figure 9). Compounds **T10** and **T11** had similar binding manners (Figure 9). The most optimal compound **T16** adopted a different binding manner, which is quite different from the other two compounds, **T11** and **T10**. In compound **T16**, the hydrogen on indole ring is close to I508 on A chain. For both compounds **T11** and **T10**, hydrogen on 1*H*-indole ring is close to T480 on C chain (Figure 3), which forms a hydrogen bond with oxygen of the carbonyl group on the backbone of T479 on C chain (Figure 3, Figure 4). Therefore, the different compounds might adopt the different manners while binding with the active site of the target Kv1.5 channel.

It was also found that some of the amino acid residues in the binding sites on the Kv1.5 channel are crucial for binding with the compound **T16** (Figure 8, Figure 9). The distances between the hydrogen atoms on I508, V512, and T480 and compound **T16** were 2.8 Å, 2.5 Å, and 2.2 Å, respectively (Figure 9A). Some new target compounds in the study potently inhibited the hKv1.5 current in a voltage- and concentration-dependent manner at a lower concentration than the reported Kv1.5 inhibitors including hesperetin (Wang et al., 2016), papaverine (Choe et al., 2003), propofol (Yang et al., 2015), and cortisone (Yu et al., 2015). Therefore, acacetin showed the distances of 2.64 Å and 0.95 Å between hydrogen atoms of I508 and V512, which was set as a comparison (Wu et al., 2011). The results promoted our understanding on the molecular interaction mechanism between the target Kv1.5 and its inhibitors (Decher et al., 2004; Decher et al., 2006); however, much remains to be done.

## Conclusion

AF is the most common clinical sustained arrhythmia; common clinical drugs have the low atrial selectivity and might cause more severe ventricle arrhythmias while stopping AF. As an anti-AF drug target with high selectivity on the atrial muscle cells, the undetermined crystal structure of the Kv1.5 channel impeded further new drug development. Herein, sixteen 3-morpholine linked aromatic amino substituted 1*H*-indoles as novel Kv1.5 channel inhibitors were designed and synthesized targeting to the simulated 3D structure of Kv1.5, which was built based on the crystal structure of its homolog Kv1.2 channel and its amino acid sequence using the molecular homology modeling technique. Compounds **T16** and **T5** (100 μM) exhibited favorable inhibitory activity against the Kv1.5 channel with an inhibition rate of 70.8 and 57.5% using a patch clamp technique. The vasodilation rates of compounds **T16** and **T4** (100 μM) reached over 90% using a Myograph. The novel Kv1.5 inhibitors would provide potential lead compounds for both anti-atrial fibrillation and anti-hypertension new drug development.

## Data Availability

The original contributions presented in the study are included in the article/Supplementary Material, further inquiries can be directed to the corresponding authors.
