# Supplementary material for: Rational Design and Synthesis of 3-Morpholine Linked Aromatic-Imino-1H-Indoles as Novel Kv1.5 Channel Inhibitors Sharing Vasodilation Effects
Source: Front Mol Biosci. 2022 Jan 24;8:805594. doi: 10.3389/fmolb.2021.805594 (PMC8819089; doi:10.3389/fmolb.2021.805594)
Supplement: Supplementary file 2 [file DataSheet2.doc]

***Supplementary Material* 2**

**Rational design and synthesis of 3-morpholine linked aromatic-imino-1*H*-indoles as novel Kv1.5 channel inhibitors sharing vasodilation effects**

**Wei Qin^1, #^, Yi-Heng Li^2, #^, Jing Tong^3, #^, Jie Wu^3^, Dong Zhao^1^, Hui-Jin Li^1^, Lu Xing^1^, Chun-Xia He^1^, Xin Zhou^1^, Peng-Quan Li^1^, Ge Meng****^3, 4^, *, Shao-Ping Wu^2^, *, Hui-Ling Cao^1, 2^, ***

*** Correspondence:** Ge Meng (email: mengge@mail.xjtu.edu.cn), Shao-Ping Wu (email: wushaoping@nwu.edu.cn), Hui-Ling Cao (email: caohuiling_jzs@xiyi.edu.cn)

^#^ The authors contributed equally to this work.

The NMR spectra of 3-(substituted aromatic imino)-1*H*-indole derivatives are listed as the following:


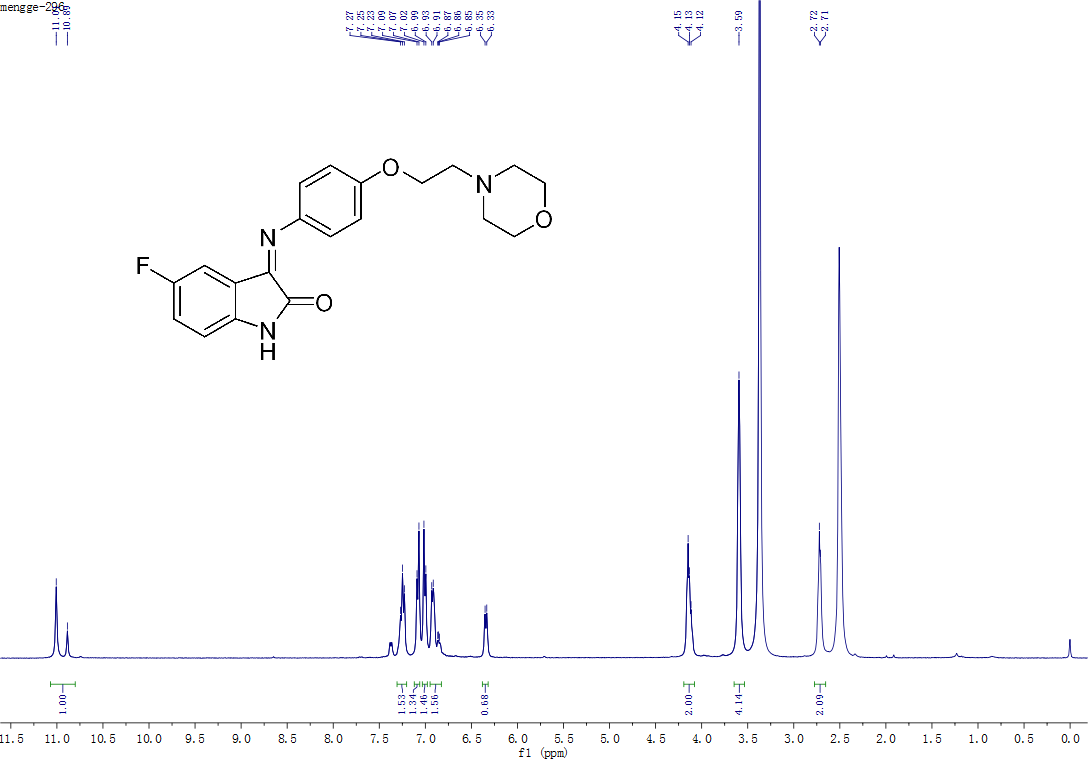


Figure 1. ^1^H NMR of the target compound **T1**


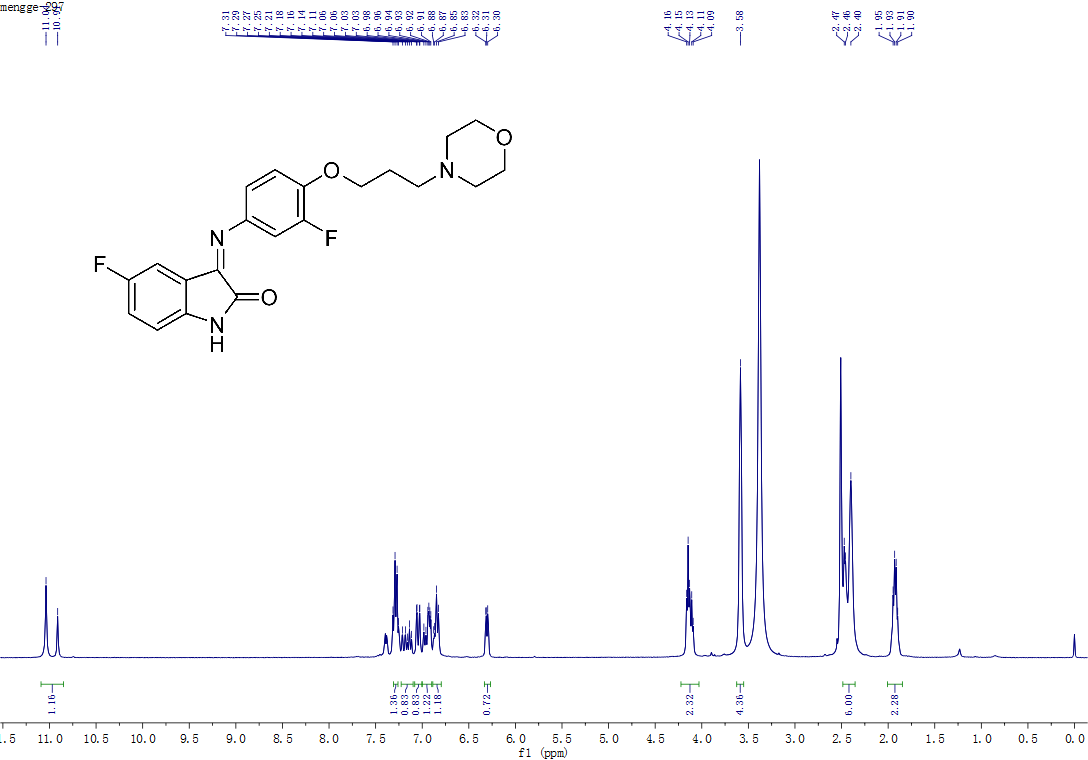


Figure 2. ^1^H NMR of the target compound **T2**


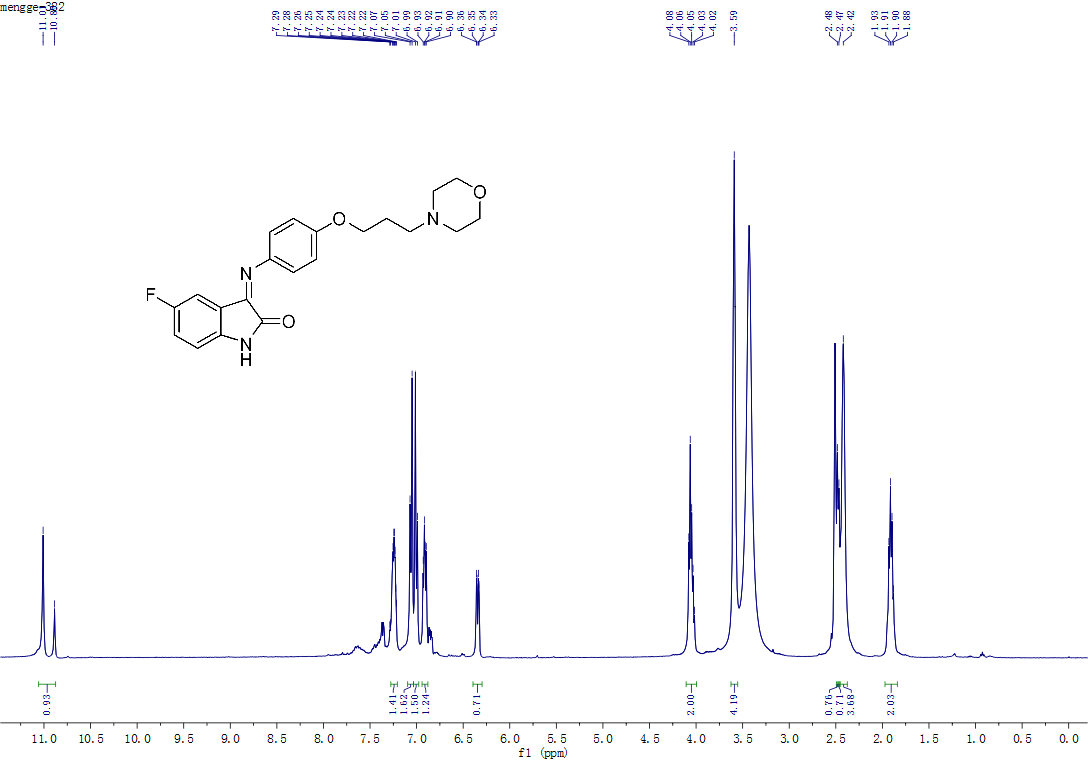


Figure 3. ^1^H NMR of the target compound **T3**


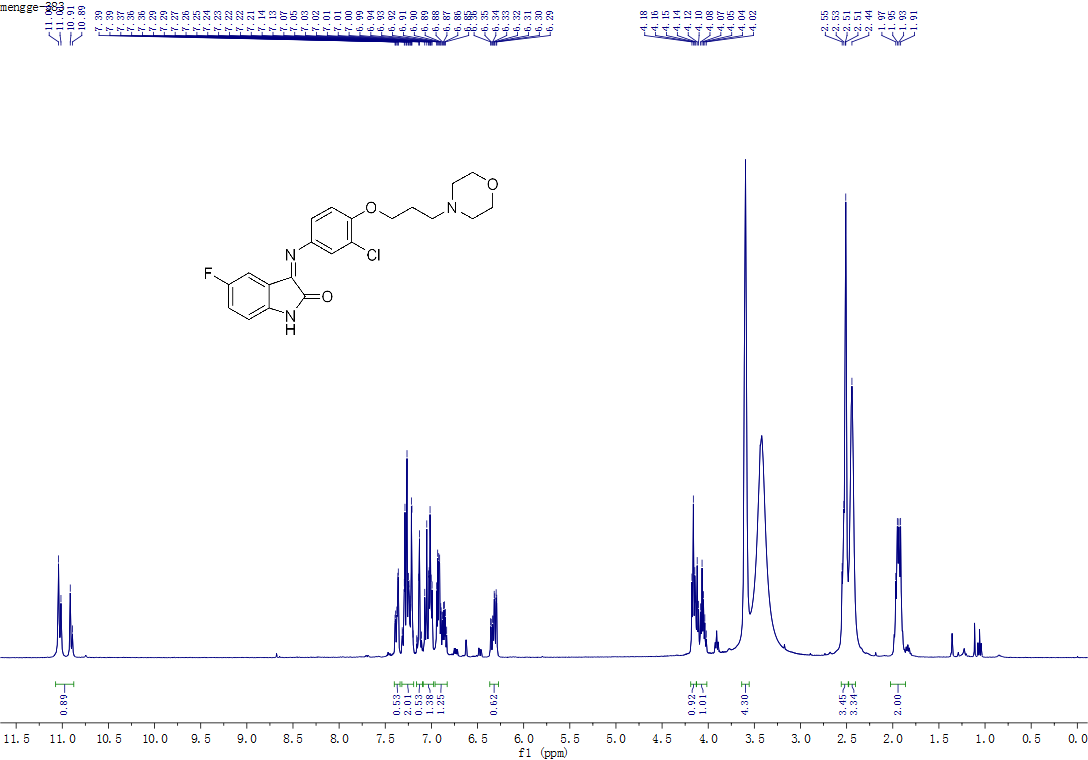


Figure 4. ^1^H NMR of the target compound **T4**


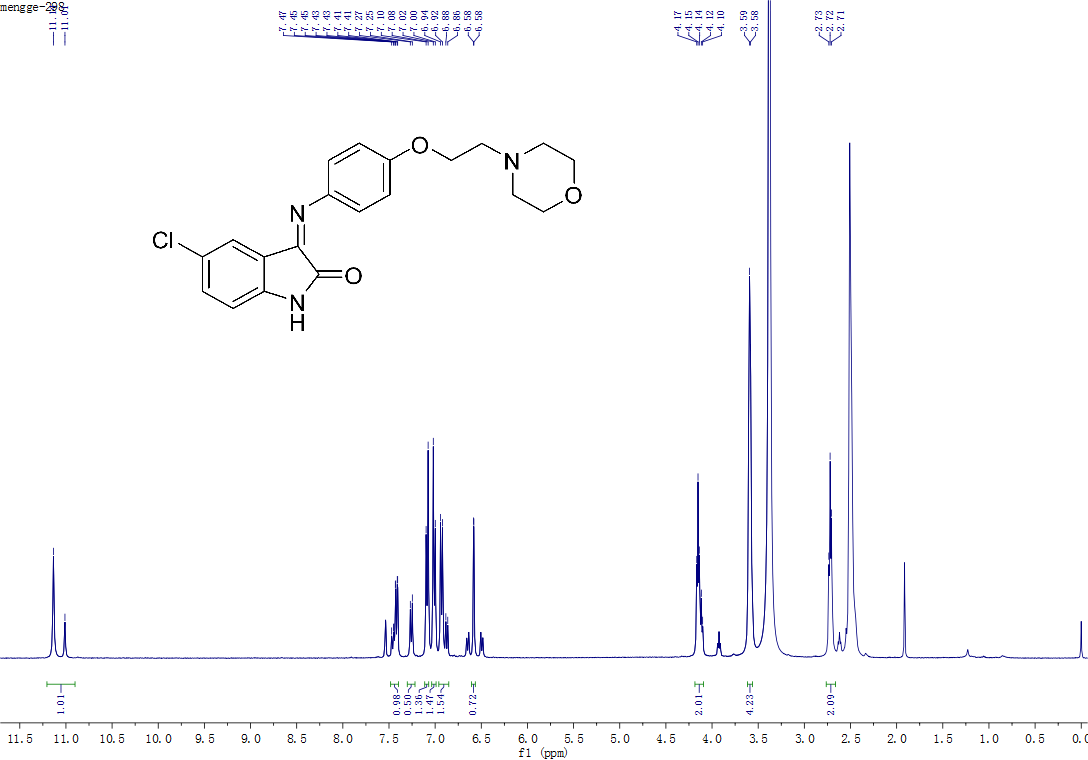


Figure 5. ^1^H NMR of the target compound **T5**


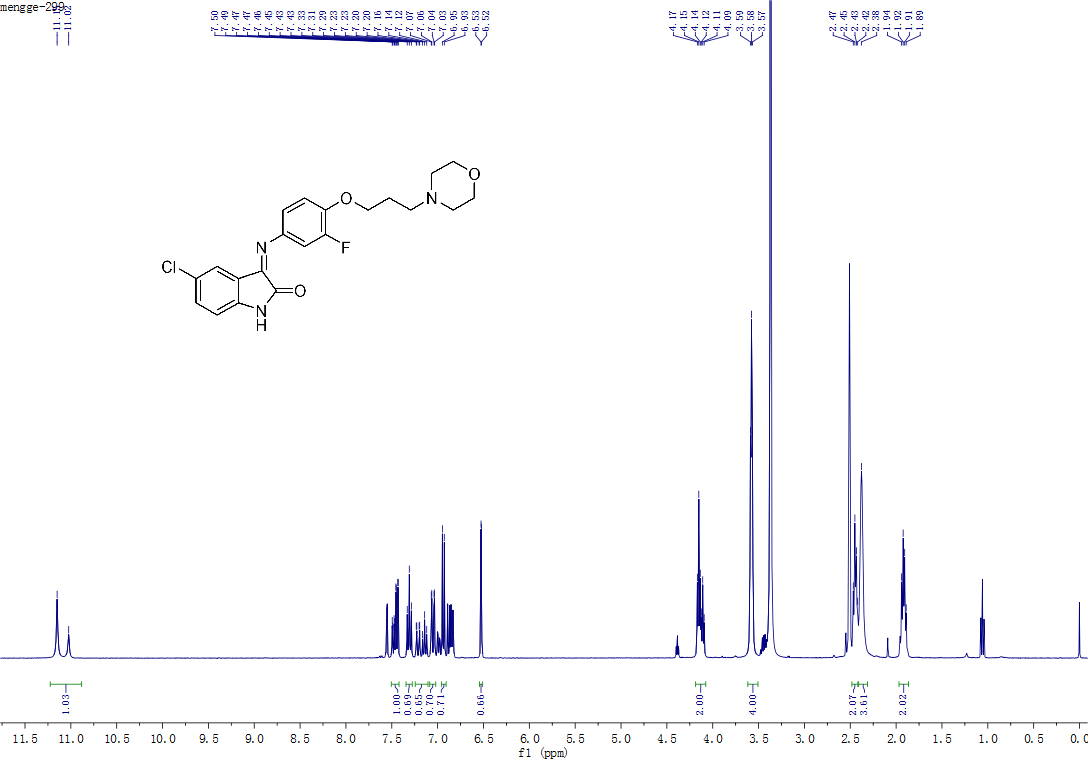


Figure 6. ^1^H NMR of the target compound **T6**


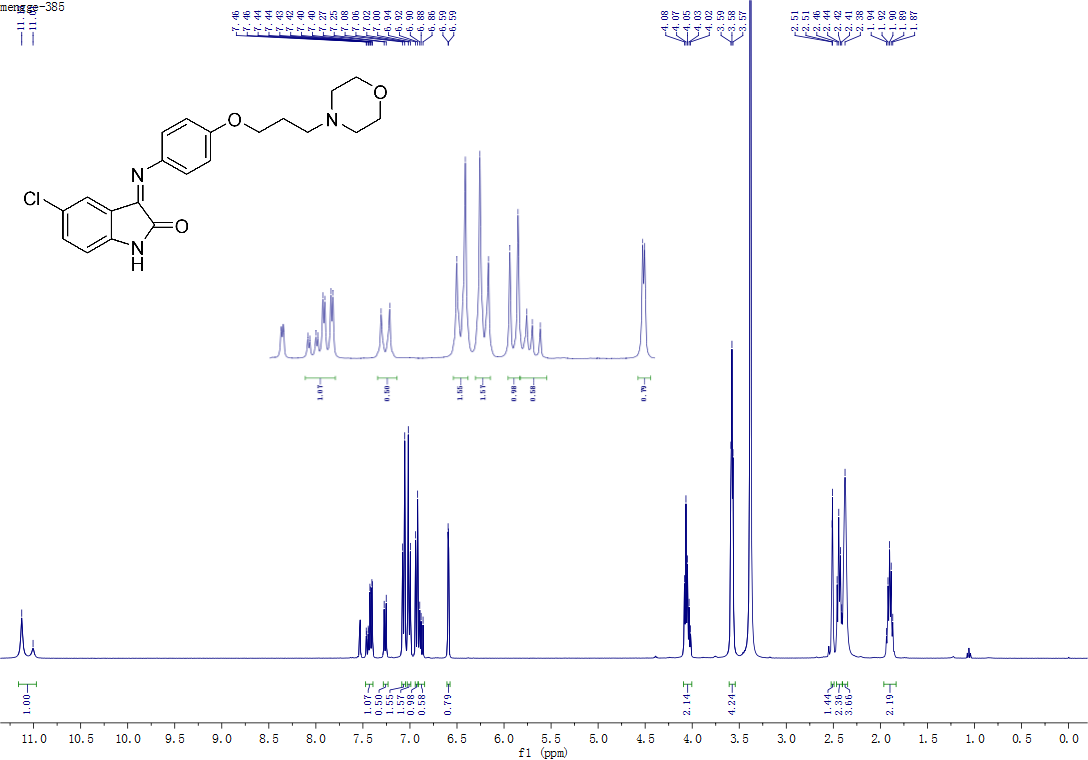


Figure 7. ^1^H NMR of the target compound **T7**


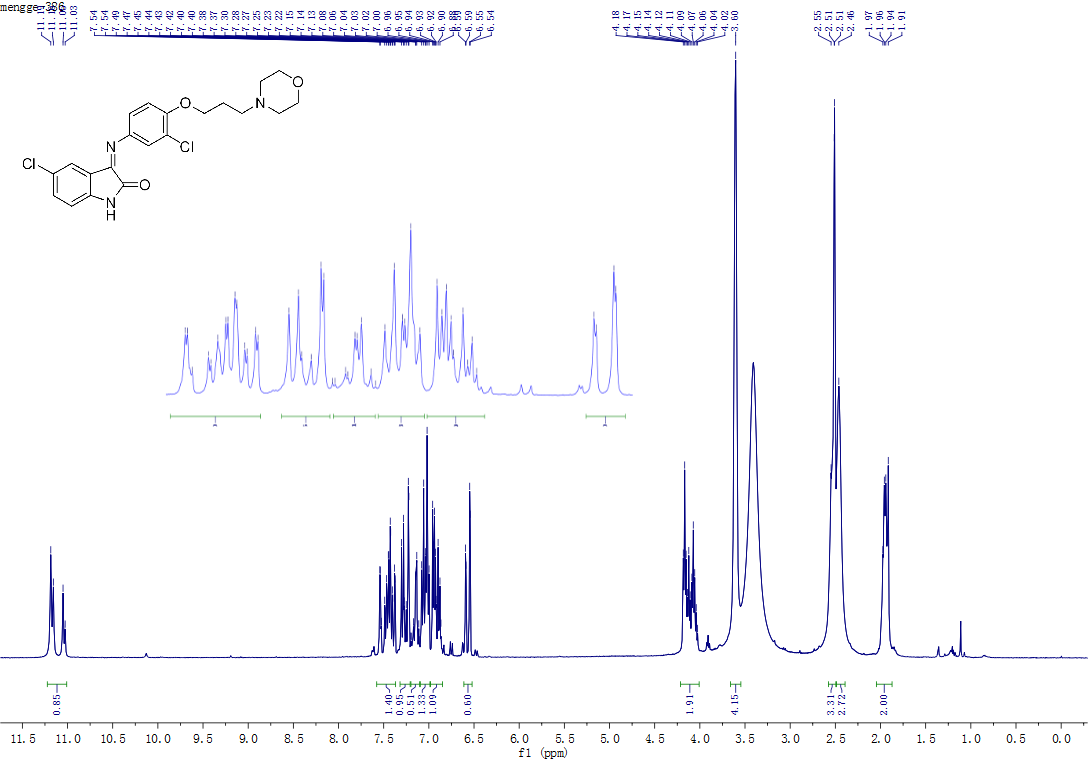


Figure 8. ^1^H NMR of the target compound **T8**


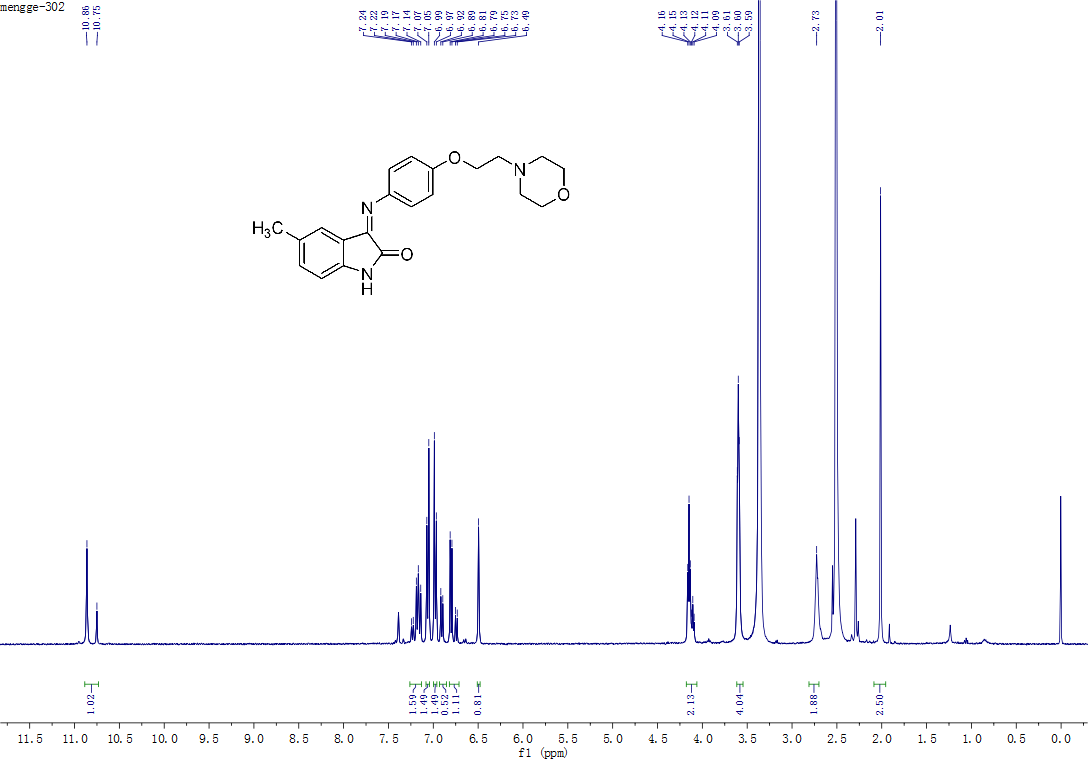


Figure 9. ^1^H NMR of the target compound **T9**


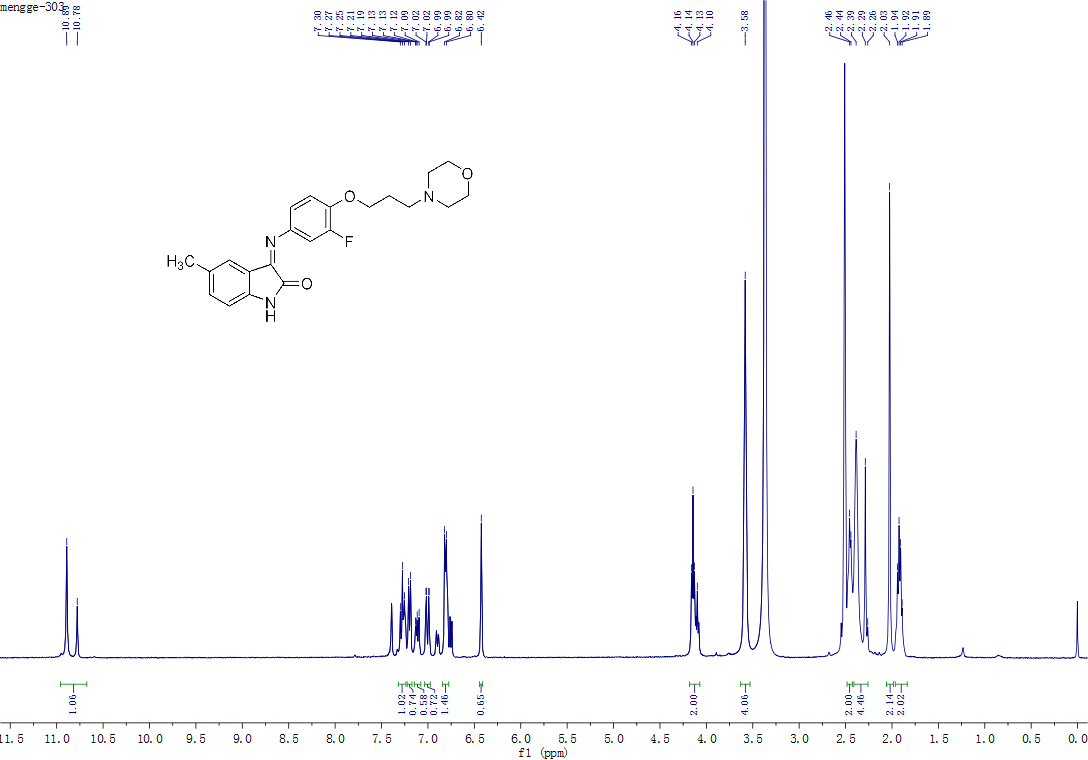


Figure 10. ^1^H NMR of the target compound **T10**


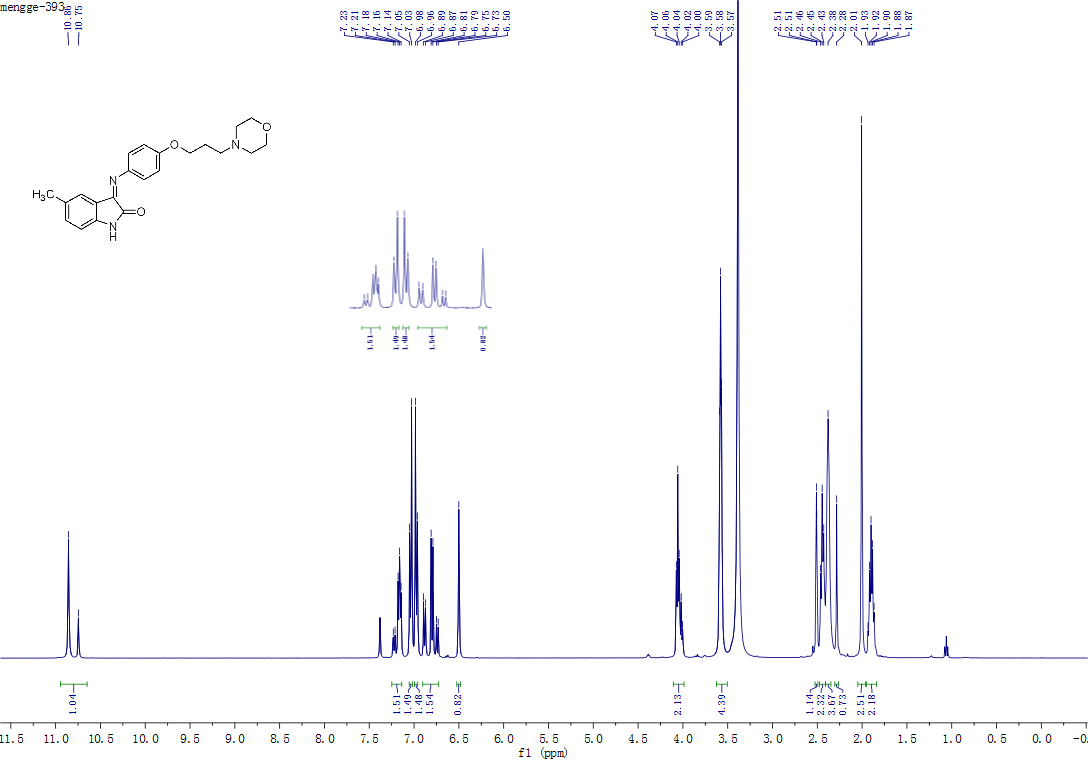
Figure 11. ^1^H NMR of the target compound **T11**


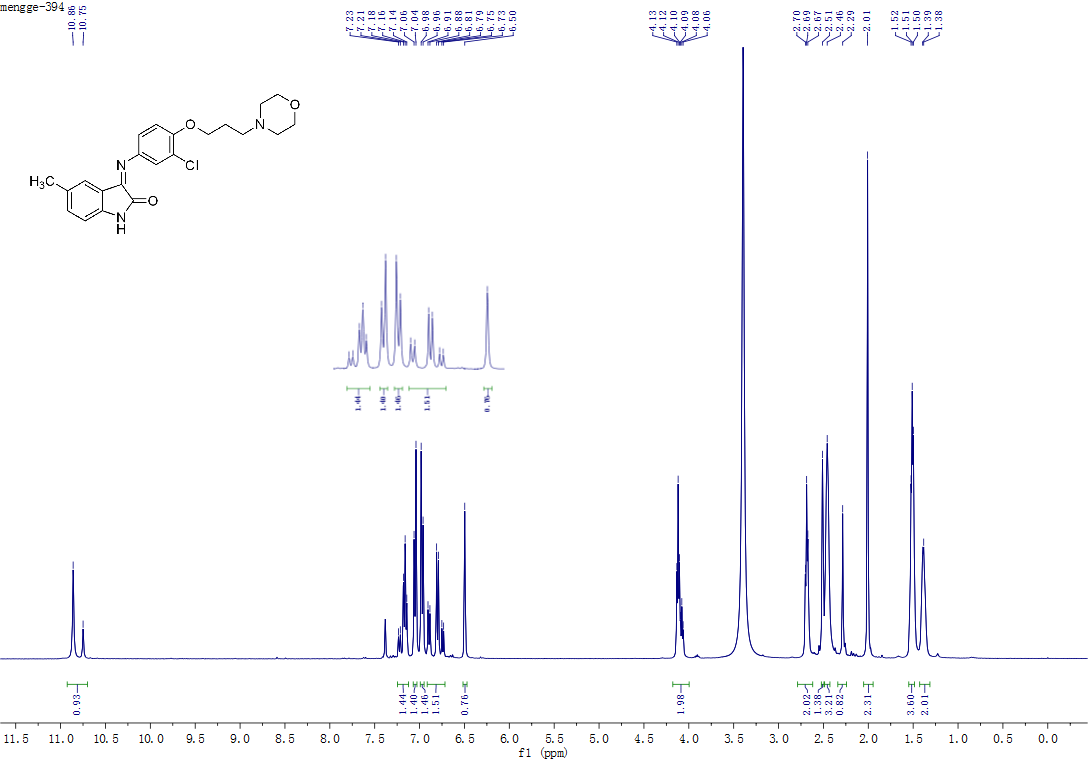


Figure 12. ^1^H NMR of the target compound **T12**


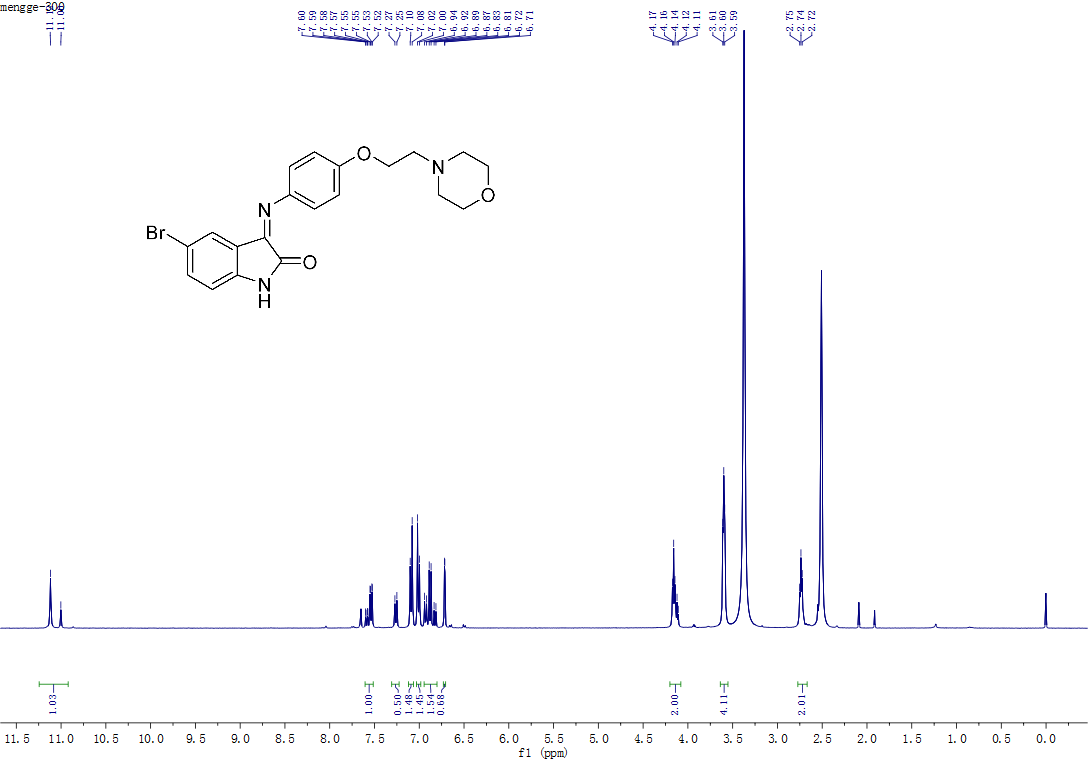


Figure 13. ^1^H NMR of the target compound **T13**


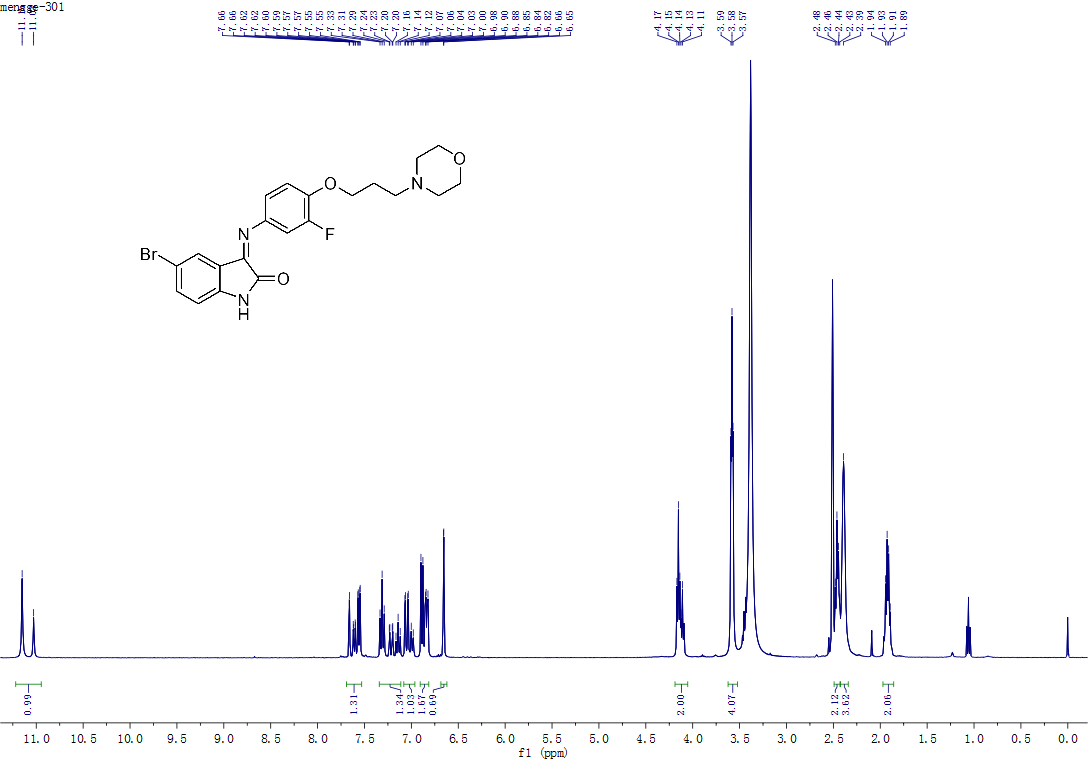


Figure 14. ^1^H NMR of the target compound **T14**


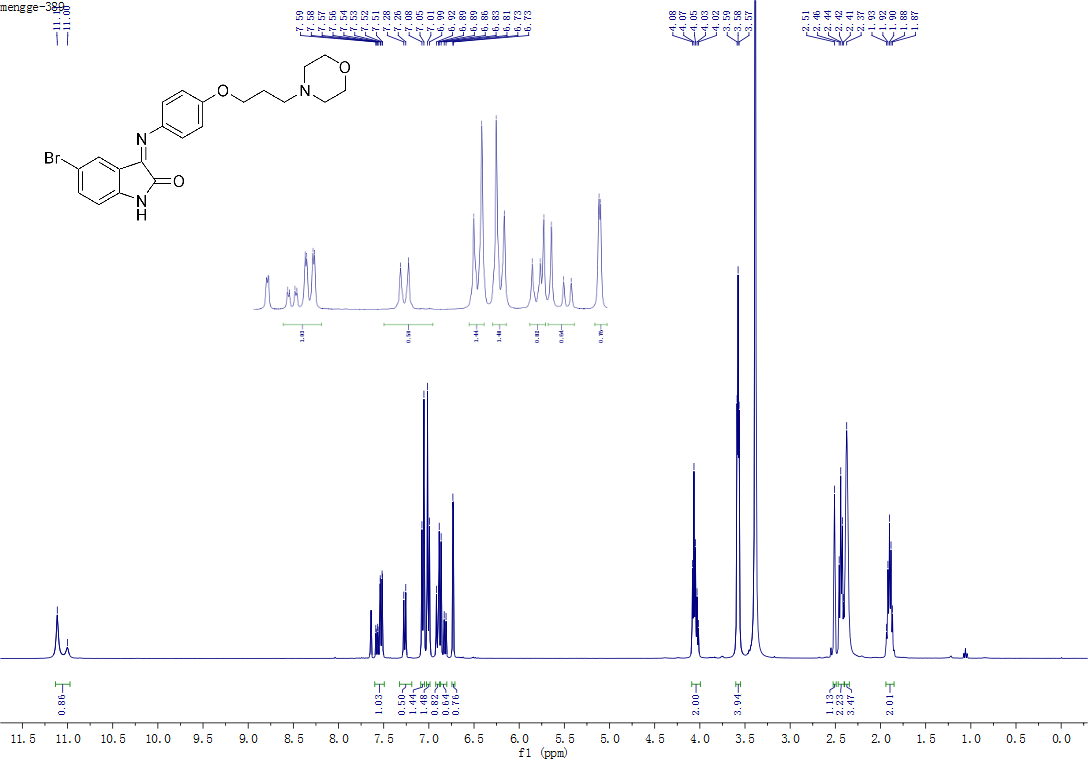


Figure 15. ^1^H NMR of the target compound **T15**


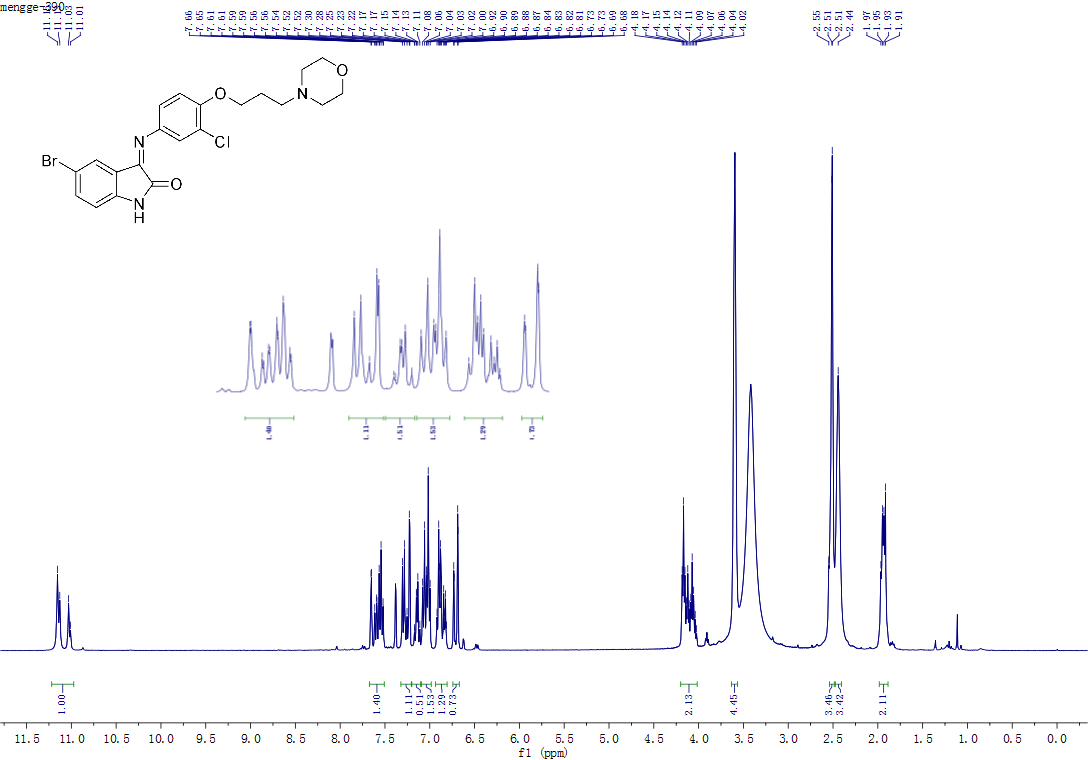


Figure 16. ^1^H NMR of the target compound **T16**
