# Supplementary material for: Rational Design and Synthesis of 3-Morpholine Linked Aromatic-Imino-1H-Indoles as Novel Kv1.5 Channel Inhibitors Sharing Vasodilation Effects
Source: Front Mol Biosci. 2022 Jan 24;8:805594. doi: 10.3389/fmolb.2021.805594 (PMC8819089; doi:10.3389/fmolb.2021.805594)
Supplement: Supplementary file 3 [file DataSheet1.docx]

Supplementary Material 1

Rational design and synthesis of 3-morpholine linked aromatic-imino-1*H*-indoles as novel Kv1.5 channel inhibitors sharing vasodilation effects

Wei Qin^1, #^, Yi-Heng Li^2, #^, Jing Tong^3, #^, Jie Wu^3^, Dong Zhao^1^, Hui-Jin Li^1^, Lu Xing^1^, Chun-Xia He^1^, Xin Zhou^1^, Peng-Quan Li^1^, Ge Meng^3, 4,^ *, Shao-Ping Wu^2,^ *, Hui-Ling Cao^1, 2,^ *

*** Correspondence:** Ge Meng (email: mengge@mail.xjtu.edu.cn), Shao-Ping Wu (email: wushaoping@nwu.edu.cn), Hui-Ling Cao (email: caohuiling_jzs@xiyi.edu.cn)

^#^ The authors contributed equally to this work.

# Supplementary Table

**Supplementary Table 1.** The yield and properties of 4-substituted-*N*-hydroxyloxime acetyl aniline and 5-substituted isatin.

| Compounds | R^1^ | Yield(%) | Form | m.p.(℃) | Compounds | R^1^ | Yield(%) | Form | m.p.(℃) |
| --- | --- | --- | --- | --- | --- | --- | --- | --- | --- |
| **2a** | 4-F | 85.6 | Yellow | 158.5~160.1 | **3a** | 4-F | 88.6 | Yellow | 219.1~220.8 |
| **2b** | 4-Cl | 80.9 | Yellow | 171.9~174.0 | **3b** | 4-l | 65.4 | Yellow | 249.2~251.4 |
| **2c** | 4-Me | 60.5 | Yellow | 154.9~155.7 | **3c** | 4-Me | 85.9 | Yellow | 185.3~186.8 |
| **2d** | 4-Br | 78.3 | Yellow | 166.8~168.3 | **3d** | 4-Br | 80.3 | Yellow | 225.8~227.1 |
